# GeneCount: genome-wide calculation of absolute tumor DNA copy numbers from array comparative genomic hybridization data

**DOI:** 10.1186/gb-2008-9-5-r86

**Published:** 2008-05-23

**Authors:** Heidi Lyng, Malin Lando, Runar S Brøvig, Debbie H Svendsrud, Morten Johansen, Eivind Galteland, Odd T Brustugun, Leonardo A Meza-Zepeda, Ola Myklebost, Gunnar B Kristensen, Eivind Hovig, Trond Stokke

**Affiliations:** 1 Department of Radiation Biology, Institute for Cancer Research, Norwegian Radium Hospital, Montebello, NO-0310 Oslo, Norway; 2 Department of Tumor Biology, Institute for Cancer Research, Norwegian Radium Hospital, Montebello, NO-0310 Oslo, Norway; 3 Department of Oncology, Norwegian Radium Hospital, Montebello, NO-0310 Oslo, Norway; 4 Norwegian Microarray Consortium, Department of Molecular Bioscience, University of Oslo, NO-0316 Oslo, Norway; 5 Department of Gynecologic Oncology, Norwegian Radium Hospital, Montebello, NO-0310 Oslo, Norway; 6 Department of Medical Informatics, University of Oslo, NP-0316 Oslo, Norway; 7 Institute of Informatics, University of Oslo, NO-0316 Oslo, Norway

## Abstract

Absolute tumor DNA copy numbers can currently be achieved only on a single gene basis by using fluorescence *in situ *hybridization (FISH). We present GeneCount, a method for genome-wide calculation of absolute copy numbers from clinical array comparative genomic hybridization data. The tumor cell fraction is reliably estimated in the model. Data consistent with FISH results are achieved. We demonstrate significant improvements over existing methods for exploring gene dosages and intratumor copy number heterogeneity in cancers.

## Background

Array comparative genomic hybridization (aCGH) is widely used for genome-wide mapping of DNA copy number changes in malignant cells [1,2]. Genetic gains and losses impact gene expression levels, and thereby promote tumor growth and progression [3-5]. Numerous clinical studies have been performed to find tumor characteristics and to classify patients with respect to their prognosis based on the copy number changes [6,7]. The usefulness of the aCGH data is limited, however, because only relative and not absolute copy numbers are achieved, making the interpretation of the data and comparisons across experiments difficult. Absolute DNA copy numbers can be obtained only on a single gene basis by the use of fluorescence *in situ *hybridization (FISH). Development of genome-wide methods for this purpose would enable generation of universal gene copy number databases of individual diseases that could be utilized more widely, as is the goal of several public repositories like the Mitelman Database of Chromosome Aberrations in Cancer [8].

The relative values achieved in aCGH experiments are influenced by the total DNA content (ploidy) of the tumor cells, the proportion of normal cells in the sample, and the experimental bias, in addition to the DNA copy numbers. The values are presented as intensity ratios between tumor and normal DNA [2]. The data are normalized so that the ratio of 1.0 is the baseline for the analysis, and corresponds to two DNA copies in near diploid (2*n*) tumors. The copy number changes are identified from the ratios deviating from the baseline, using statistical methods for ratio smoothing and breakpoint detection [9-12]. To assign an absolute copy number to each ratio level identified by the statistical analysis and thereby score genetic aberrations are, however, challenging. In aneuploid tumors with gross alterations in the DNA content, the baseline represents a copy number other than 2, like 3 or 4 in tri- or tetraploid tumors, or a non-integer value when the DNA content differs from *n*, 2*n*, 3*n*, ... m*n *[13]. The presence of normal cells within the sample and experimental bias reduce the ratio dynamics. Moreover, in many tumors, several subpopulations of malignant cells with different genetic characteristics exist, leading to intratumor heterogeneity in the DNA copy numbers [14-16] and increased complexity in the data. Unreliable results occur, therefore, when common ratio levels are used to score gains and losses in tumors with different ploidy and normal cell content.

The confounding effect caused by normal cells within tumor samples is recognized as a problem in aCGH analyses and has been handled by excluding low purity samples [17,18] or correcting the ratio levels based on histological examination of tumor sections [6]. The latter approach is not satisfactory because only the proportion of connective tissue surrounding the tumor parenchyma, and not the infiltrating immune cells, is precisely quantified. Moreover, the measurements cannot be performed on exactly the same tissue as used in the aCGH experiment and may, therefore, not be representative. A model including the CGH ratios, ploidy, and experimental bias has been proposed for estimation of absolute DNA copy numbers in tumor cell lines [19]. To our knowledge, no method exists that also considers the normal cell content and, thus, is suited for analyses of clinical tumor samples.

We here present a new model, GeneCount, where the proportion of normal cells is estimated and corrected for and possible intratumor heterogeneity in DNA copy numbers is considered. Inputs to our model are the DNA index (*DI*, where *DI *= 1/2·tumor ploidy), tumor cell fraction, experimental bias, and aCGH ratios. Predetermined measures of tumor ploidy, determined either by flow or image based cytometry, are needed. The tumor cell fraction can be determined by, for example, flow cytometry on the same part of the sample as used in the aCGH experiment. In cases of unknown normal cell content, the tumor cell fraction is estimated in the model. The experimental bias is determined from the X-chromosome ratio in aCGH experiments where male and female DNA is compared. Smoothed ratio levels from any existing statistical analysis tools for breakpoint detection can be used.

We show that the model enabled automatic and genome-wide calculation of DNA copy numbers from aCGH data of both hematopoietic and solid tumors. The feasibility of GeneCount was demonstrated by analysis of 94 lymphomas, for which the DNA index and tumor cell fraction had been determined by use of flow cytometry and an extensive exploration of DNA copy numbers had been performed by the use of FISH in previous studies [20-25]. The GeneCount results, both based on the pre-determined tumor cell fraction and that determined by the model, were compared with the FISH data of 362 genes with and without gains and losses, showing 97% consistency in both cases. In particular, we explored the copy numbers achieved in the t(14;18) translocated chromosomal region involving *BCL2*. We further demonstrated the potential of GeneCount in analysis of solid tumors without pre-determined tumor cell fractions by relating the copy number of selected genes in 93 cervical cancers to gene expression and treatment outcome. By use of GeneCount we obtained a higher sensitivity in detecting cervix tumors with copy number changes than was obtained in analysis based directly on the ratio levels. Finally, we identified intratumor heterogeneity of DNA copy numbers in the lymphomas and cervical cancers, and showed how this information could be used to draw conclusions about the evolution of the genetic aberrations in the tumors. GeneCount was implemented in a software package to be used downstream of statistical methods for breakpoint detection, and results based on both the GLAD and CGH-Explorer packages are presented [9,11]. We supply our method through the open-source and free web-based database BioArray Software Environment (BASE) [26].

## Results

### Basis of GeneCount

**Figure 1:**
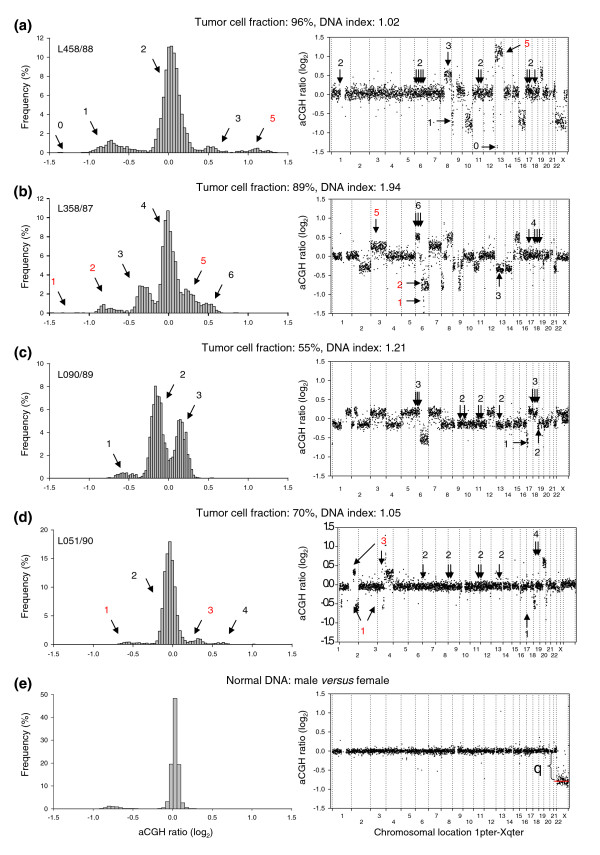
Illustration of the stepwise increase in aCGH ratios with increasing DNA copy number. Frequency histograms (% array probes) of aCGH ratios (left panels) and plot of aCGH ratio versus chromosomal location (right panels) are shown for a lymphoma with a DNA index (*DI*) of **(a) **1.02, **(b) **1.94, **(c) **1.21, and **(d) **1.05, and **(e) **for normal DNA comparing male and female. The tumor cell fraction, measured by flow cytometry, is indicated for each tumor. DNA copy numbers estimated by GeneCount are marked; those in black were consistent with FISH data, whereas those in red have not been subjected to FISH measurements in the specific tumors shown. The arrows in the right panels point to the locations of the FISH probes. At a *DI *close to 1 and 2 (a,b,d,e) the ratio distribution shows a major peak at a median log_2 _value of approximately zero, representing the most frequent DNA copy numbers of 2 and 4, respectively. At a *DI *of 1.21 (c) the baseline at a log_2 _ratio of 0 represents a number between 2 and 3 DNA copies. Note the smaller increase in the ratios with increasing DNA copy number at a tumor cell fraction of 70% (d) than of 96% (a). In (e), determination of the dynamic factor, *q*, as the absolute value of the X-chromosome log_2 _ratio level is indicated.

Our model utilizes the fact that the normalized aCGH ratio increases with increasing DNA copy number in a stepwise manner, where the step size is dependent on the *DI*, the tumor cell fraction, and the experimental bias (Figure 1). In near diploid tumors (*DI *= 1) without a contribution from normal cells or affected by experimental bias, an increment of 1 in the copy number increases the ratio by a value of 0.5, leading to a normalized ratio of 0.5, 1, 1.5, 2, and so on (-1, 0, 0.69, 1 on a log_2 _scale) for a copy number of 1, 2, 3, and 4, respectively (see Equation 2 in Materials and methods). The corresponding increase in tetraploid tumors (*DI *= 2) is 0.25, whereas an increase between 0.25 and 0.5 occurs in tumors with a *DI *between 1 and 2. Baseline, at a log_2 _ratio of 0, corresponds to 2, 3, and 4 DNA copies in near diploid (Figure 1a), triploid, and tetraploid (Figure 1b) tumors, respectively. For *DI*s between 1 and 1.5 or between 1.5 and 2, baseline represents a copy number between 2 and 3 (Figure 1c) or between 3 and 4. The presence of normal cells within the tumor sample reduces the increase in aCGH ratio with incremental copy number (Equation 3), as can be seen when comparing the ratios of two near diploid lymphomas with different tumor cell fractions (Figures 1a,d). Using common ratio levels for scoring gains and losses in tumors like those presented in Figures 1a-d leads, therefore, to different results with respect to copy number changes.

A further reduction in the ratio dynamics occurs due to experimental bias (Equation 4). The bias, as represented by the dynamic factor, *q*, can be determined from control experiments, where normal DNA from males and females is cohybridized (Figure 1e). Theoretically, the X-chromosome ratio is 0.5 (-1 on a log_2 _scale), but the experimental bias reduces the ratio dynamics, leading to a ratio level closer to zero. The absolute value of the log_2_-transformed ratio level was used as a measure of *q *(Figure 1e). This value differed little among the slide series used here, ranging from 0.75-0.85 with a mean ± standard deviation of 0.80 ± 0.04 based on 8 control experiments. A *q-*value of 0.8 and range of 0.7-0.9 was used in the GeneCount calculations in the cases of known and unknown tumor cell fraction, respectively.

To enable automatic calculation of the copy number associated with each array probe, we implemented GeneCount in a program to be run on top of statistical analysis packages for aCGH ratio smoothing and breakpoint detection (Additional data file 1). A separate algorithm was developed for samples with unknown tumor cell fraction, where the fraction was estimated based on two ratio levels and *DI *(panel B in Additional data file 1), as described in Materials and methods. One decimal was included in the calculated DNA copy numbers when evaluating the results in comparison with FISH data. Otherwise, the numbers were rounded off to the nearest integer values.

### GeneCount copy numbers in comparison with FISH data

We compared the GeneCount results of 94 lymphomas with previously published FISH data from the same tumors [20-25]. The FISH probes were located at chromosomal regions with frequent copy number changes (Figure 1 and Additional data file 2), and copy numbers within the range of 0-8 had been measured. The *DI*s, ranging from 0.95-2.23, and the tumor cell fractions, ranging from 27% to 98%, were used as inputs to GeneCount, together with the smoothed aCGH ratios from the GLAD and CGH-Explorer packages. CGH-Explorer applied a more extensive ratio smoothing than GLAD, and this led occasionally to differences in the ratio levels and breakpoint detection between the two programs.

#### GeneCount with known tumor cell fraction

**Figure 2:**
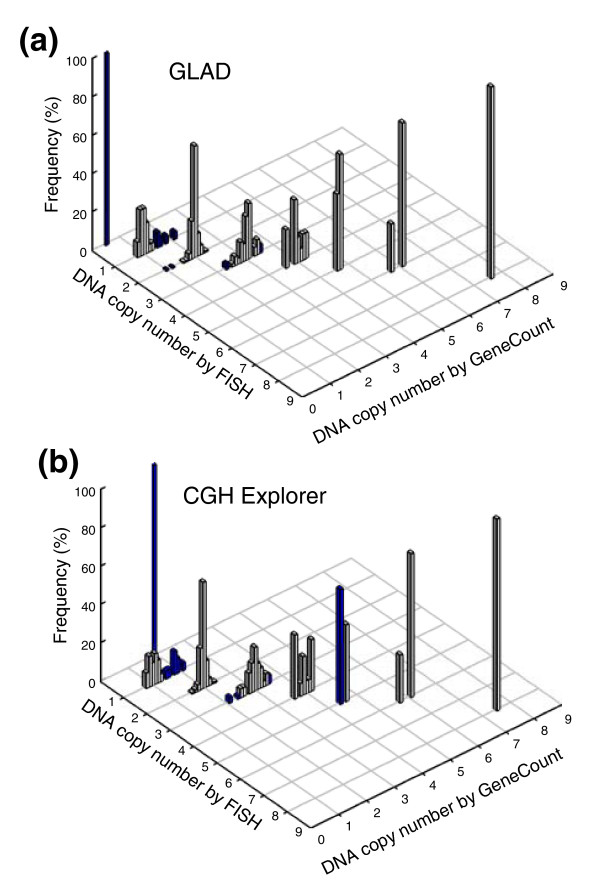
GeneCount calculations with known tumor cell fraction. DNA copy number calculated by GeneCount is plotted against the corresponding FISH result for 9 genes in 94 lymphomas. The smoothed aCGH ratios from **(a) **GLAD and **(b) **CGH-explorer, a *q*-value of 0.8, and a *DI* and tumor cell fraction determined by flow cytometry were inputs to GeneCount. Grey and blue columns represent GeneCount results that were consistent and inconsistent with the FISH data, respectively, after rounding off the GeneCount number to the nearest integer value. Frequency distributions are shown for each copy number, containing 1, 25, 246, 66, 15, 5, 4, and 1 value at a FISH copy number of 0, 1, 2, 3, 4, 5, 6, and 8, respectively.

In most cases, we found an excellent agreement between the DNA copy number determined by GeneCount and FISH, regardless of whether GLAD or CGH-Explorer was used for breakpoint detection (Figure 2). The correlation between the data sets was considerably better than when the ratio levels were used in the comparison (Additional data file 3). Based on GLAD, 350 out of 362 GeneCount values were consistent with the FISH data (97%), whereas the corresponding number based on CGH-Explorer was 340 out of 362 (94%) (Figure 2). The few discrepancies between the GeneCount and FISH results occurred mainly for two reasons. First, GLAD and/or CGH-Explorer failed to detect the ratio change of some of the genes that had a copy number change by FISH (panel A in Additional data file 4). Second, the ratio level, and therefore the copy number, was inaccurately determined for some aberrations involving only a few array probes (panel B in Additional data file 4). This was primarily the case for aberrations with less than three probes, like the homozygote deletion involving two probes that covered *RB1 *in one of the tumors (FISH copy number of 0 in Figure 2 and panel B in Additional data file 4). The discrepancies between the GeneCount and FISH data were related, therefore, to the software used for breakpoint detection and not due to errors in the GeneCount algorithm.

#### GeneCount with unknown tumor cell fraction

**Figure 3:**
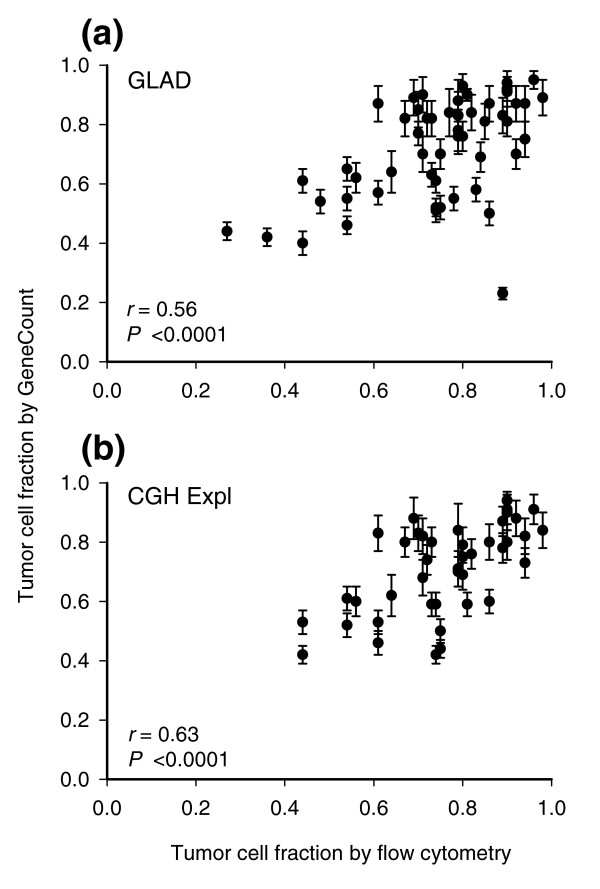
GeneCount estimations of tumor cell fraction. Tumor cell fraction of lymphomas estimated by GeneCount is plotted against tumor cell fraction measured by flow cytometry. Each point represents mean ± standard deviation based on the values achieved for *q *within the range 0.7-0.9. The smoothed aCGH ratios from **(a) **GLAD and **(b) **CGH-explorer, the *q *range 0.7-0.8, and a *DI *determined by flow cytometry were inputs to GeneCount. The calculations were based on 55 (a) and 43 (b) tumors for which suitable ratio levels for the calculations existed. Correlation coefficients and *P*-values from Pearson product moment correlation analyses are indicated.

The tumor cell fraction could be estimated for 55 and 43 out of 94 lymphomas based on GLAD and CGH-Explorer, respectively. The remaining tumors lacked aberrations or two different ratio levels that could be used for the estimation (Materials and methods). The estimated tumor cell fractions correlated significantly with those measured by flow cytometry (Figure 3). Moreover, the estimates had a coefficient of variance (CV) of less than 11% (Figure 3), and were therefore fairly stable. The mean *q*-value determined in the calculation differed little across the tumors, ranging from 0.73-0.84 (GLAD) and 0.74-0.82 (CGH-Explorer) (data not shown).

**Figure 4:**
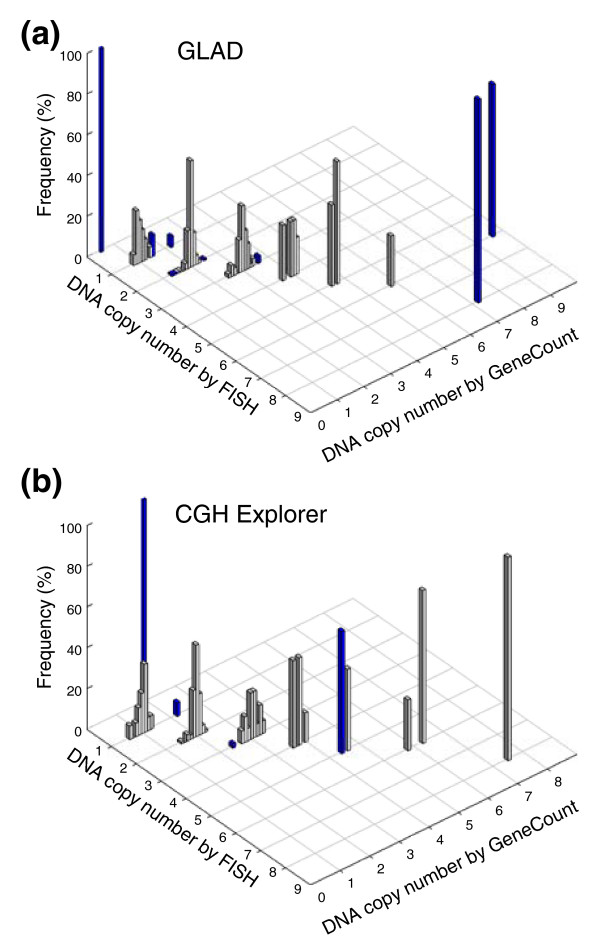
GeneCount estimations with unknown tumor cell fraction. DNA copy number calculated by GeneCount, using a *q*-value within the range 0.7-0.9, a *DI *determined by flow cytomery, and the tumor cell fraction estimated by GeneCount in Figure 3, is plotted against the corresponding FISH result for 9 genes in **(a) **55 and **(b) **43 lymphomas. The smoothed array CGH ratio derived from GLAD and CGH-explorer was used in (a) and (b), respectively. Grey and blue columns represent GeneCount results that were consistent and inconsistent with the FISH data, respectively, after rounding off the GeneCount value. Frequency distributions are shown for each copy number, containing 1, 19, 134, 56, 11, 5, 4, and 1 value at a FISH copy number of 0, 1, 2, 3, 4, 5, 6, and 8, respectively, based on GLAD. The corresponding numbers based on CGH Explorer were 1, 15, 98, 48, 7, 5, 4, and 1.

The consistency between the GeneCount and FISH data (Figure 4) was comparable to when the known tumor cell fraction was used (Figure 2) and much better than when the ratio levels and FISH data were compared (Additional data file 3). Based on GLAD, 218 out of 231 DNA copy numbers were in agreement with the FISH data (94%), whereas the corresponding numbers based on CGH-Explorer were 173 out of 179 (97%) (Figure 4). Most differences between the GeneCount and FISH results occurred for the same reasons as when the known tumor cell fraction was used (Additional data file 4). Additionally, a discrepancy was seen for some of the highest copy numbers based on GLAD (Figure 4), due to a large discrepancy between the estimated and measured tumor cell fraction in one of the cases (Figure 3a).

#### DNA copy numbers in translocated chromosomal regions

**Figure 5:**
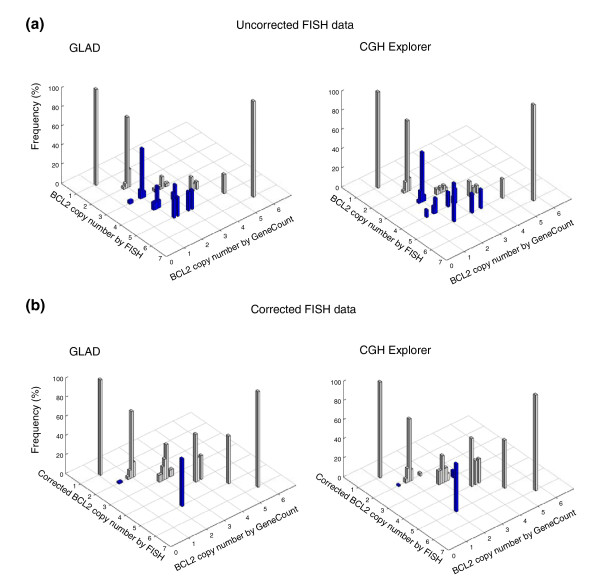
GeneCount estimations in the t(14;18) translocated region involving *BCL2*. *BCL2 *copy number estimated by GeneCount, using a *q*-value of 0.8 and a *DI *and tumor cell fraction determined by flow cytometry, is plotted against the corresponding FISH result in 94 lymphomas. The smoothed array CGH ratios derived from GLAD and CGH-explorer were used in the left and right panels, respectively. Grey and blue columns represent GeneCount calculations that were consistent and inconsistent with the FISH measurements, respectively, after rounding off the GeneCount value. **(a) **Uncorrected FISH data are plotted; **(b) **these data were corrected as described in [22]. Frequency distributions are shown for each copy number, containing 1, 38, 33, 13, 5, and 1 value for a red spot FISH copy number of 1, 2, 3, 4, 5, and 6. The corresponding number of measurements for the corrected FISH data of 1, 2, 3, 4, 5, and 6 were 1, 69, 14, 4, 2 and 1.

The relationship between the GeneCount estimates and FISH data in translocated chromosomal regions was explored by using *BCL2*, which is involved in the translocation t(14;18) in lymphomas, as an example. The aCGH probe covering *BCL2 *is located telomeric of the breakpoint. The aCGH data and GeneCount results of *BCL2 *were therefore not affected by the translocation. For FISH analysis, we selected a *BCL2 *probe covering the breakpoint. The probe signal was split in tumors with translocation, leading to a signal from both der(14)t(14;18) and der(18)t(14;18), although *BCL2 *is located on the former chromosome. The FISH signal was therefore higher than the actual *BCL2 *copy number, and differed from the GeneCount result in all 38 tumors with translocation (Figure 5a). After recalculating the FISH copy numbers as described [22], the consistency in the data was excellent, except in one case at a corrected FISH value of five copies (Figure 5b). This discrepancy was due to failure of GLAD and CGH-Explorer in detecting a narrow amplicon involving *BCL2 *(panel C in Additional data file 4).

### GeneCount analysis of solid tumors

The feasibility of our method for analysis of solid tumors without information of tumor cell fraction was explored in 99 cervical cancers, for which the *DI *ranged from 1.00-3.16. The tumor cell fraction could be estimated for 93 and 89 tumors based on GLAD and CGH-Explorer, respectively, fulfilling the requirements for this estimation (Materials and methods). The tumor cell fractions were poorly correlated with the values determined by analysis of histological sections (Additional data file 5). In most cases, the histology result was higher than the GeneCount estimate, probably because immune cells infiltrating the tumor parenchyma were not properly quantified by the histological examination. In a few cases, however, the histology result was higher, probably reflecting that different parts of the sample were used in the aCGH and histology analyses. The tumors for which the tumor cell fraction could be estimated by GeneCount were included in the further analyses.

A higher number of genetic aberrations were generally found in the cervical cancers than in the lymphomas. High level amplifications with more than 2.5-fold increases in gene dosage (that is, copy number, *N*, relative to total DNA content given by two times the DNA index (*N*/(2.*DI*)), were found in about half of the tumors and most frequently on chromosomes 5p and 11q. GeneCount analysis showed copy numbers within the range of 5-80 in these regions, which were often surrounded by gains at lower levels.

**Figure 6:**
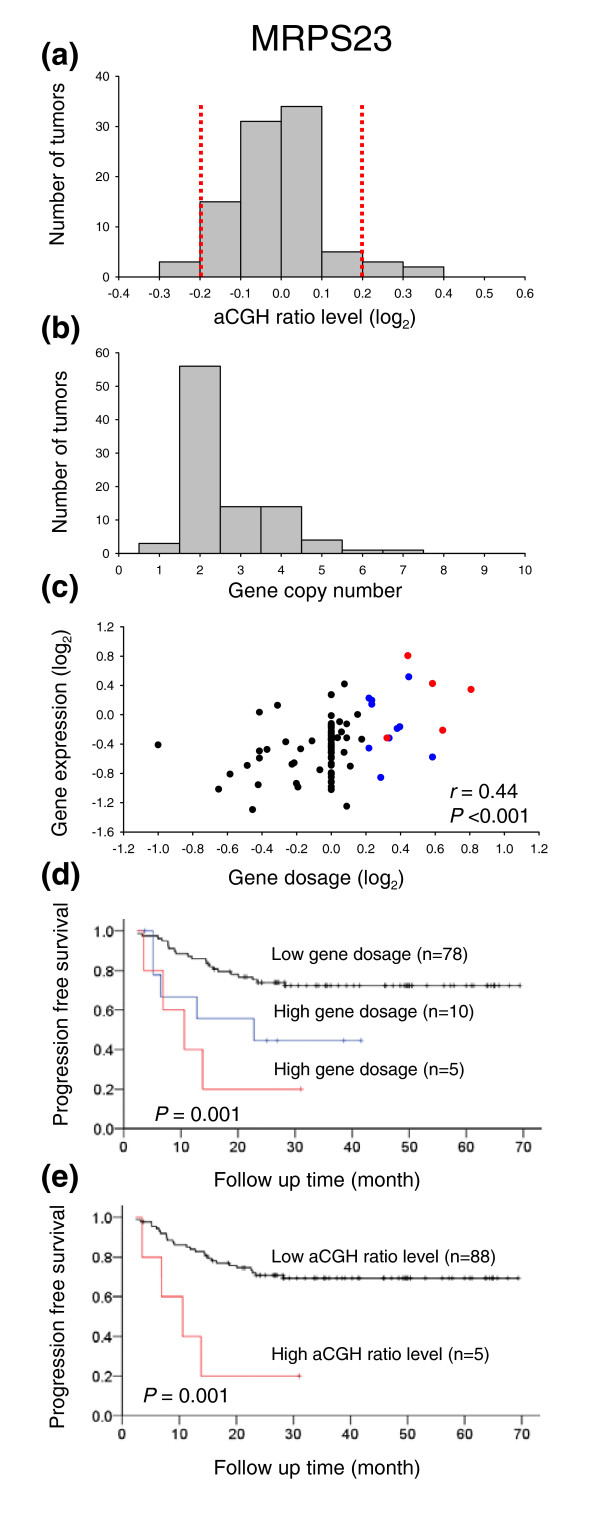
GeneCount analyses in cervical cancers. **(a) **Frequency histogram (number of tumors) of smoothed aCGH ratios (GLAD) for *MRPS23 *(BAC clone ID RP11-19F16). Dotted lines indicate the cut off ratio levels of ± 0.2, identifying 5 tumors with genetic gain and 3 tumors with loss. **(b) **Frequency histogram (number of tumors) of *MRPS23 *copy number calculated by GeneCount. The GLAD ratio levels, the *DI *measured by flow cytometry, and the tumor cell fraction estimated by GeneCount were used in the calculation. Similar results were achieved based on the CGH-Explorer ratio levels. **(c) **Plot of gene expressions against gene dosage; that is, the *MRPS23 *copy number divided by the total DNA content (*N*/(2·*DI*)). Increased gene dosage with more than 15% of the total DNA content (log_2 _transformed gene dosage of at least 0.2) were seen in 15 tumors (red and blue symbols). Red symbols represent the five tumors with gain in (a), whereas blue symbols represent the remaining ten tumors with increased gene dosage that were not identified in (a). The correlation coefficient and *P*-value from Pearson product moment correlation analysis are indicated. **(d) **Kaplan Meier analysis based on GeneCount results for *MRPS23*. Plots of the survival probability are shown for 5 patients with high gene dosage in (c), who also had gain in (a) (red line), 10 patients with high gene dosage in (c) and without gain in (a) (blue line), and 78 patients with low gene dosage in (c). **(e) **Kaplan Meier analysis based on the *MRPS23 *ratio levels. The survival probability of 5 patients with gain in (a) (red line) and 88 patients without gain in (a) (black line) is plotted. Only five high risk patients were identified in (e), whereas ten more patients were identified by GeneCount in (d). *P*-value in log-rank test is indicated in (d,e). Panels (a,b,d) are based on 93 tumors, for which the tumor cell fraction could be estimated by GeneCount. Panel (c) is based on 89 of these tumors, for which both DNA copy number and gene expression were available.

The GeneCount results were compared with the outcomes of existing analysis methods, where gains and losses were scored from the smoothed ratio levels and breakpoints obtained by GLAD and CGH-Explorer. The log_2 _transformed ratio levels of ± 0.2 (that is, approximately two times the ratio standard deviation (Additional data file 6)) were applied as cut-off levels for scoring aberrations. We selected genes that were shown to be affected by gains and losses in previous studies on a subgroup of the patients [27]. Some of the genes showed only a small variation in the aCGH ratios, often within the level of ± 0.2, and only a few tumors with aberrations were identified (Figure 6a and panel A in Additional data file 7). A higher number of patients with changes in gene copy numbers and in the corresponding gene dosages were identified with GeneCount, using the cut-off levels of ± 0.2 for scoring gene dosage changes (Figure 6b,c and panels B and C in Additional data file 7). The gene dosage correlated significantly with gene expression (Figure 6c and panel C in Additional data file 7), making the copy number changes determined by GeneCount plausible.

The copy number changes of *MRPS23 *have previously been shown to correlate with survival probability [27]. Survival analysis based on the GeneCount data of *MRPS23 *identified more patients with poor outcome than the corresponding analysis based on ratio levels (Figure 6d,e). Hence, 15 high risk patients were identified based on the GeneCount results, whereas only 5 patients were classified with high risk based on the ratio levels. Nine of the ten patients that were not identified based on ratio levels (blue curve in Figure 6d) had aneuploid tumors with a DNA index ranging from 1.10-1.92. The remaining diploid tumor had a relatively low tumor cell fraction of 23%.

### Intratumor heterogeneity in DNA copy numbers

**Figure 7:**
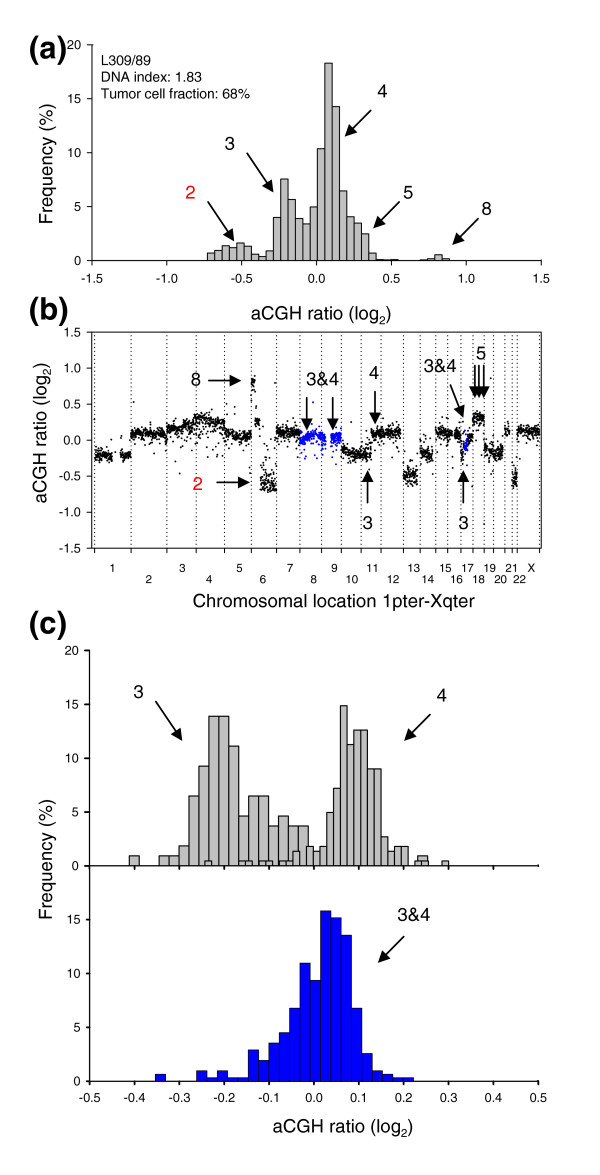
GeneCount identification of DNA copy number heterogeneity within tumors. **(a) **Frequency histogram (% array probes) of aCGH ratios in a heterogeneous lymphoma, including data for the entire genome. **(b) **aCGH ratios are plotted against chromosomal location, showing the heterogeneous regions on chromosomes 8, 9, and 17 with a DNA copy number of 3&4 in blue. **(c) **Frequency histogram (% array probes) of aCGH ratios for two homogeneous DNA regions with a copy number of 3&4 (upper panel) and the heterogeneous region depicted in (b) with a copy number of 3&4 (lower panel). The ratio distributions of copy number 3, 4, and 3&4 were significantly different (*p *< 0.001, ANOVA). DNA copy numbers estimated by GeneCount from the *DI *and tumor cell fractions measured by flow cytometry are marked; those in black were consistent with FISH experiments, whereas those in red have not been subjected to FISH measurements in the specific tumors shown. The arrows in (b) point to the locations of the FISH probes. Note that the 3&4 copy number of the heterogeneous region has been confirmed with FISH.

Some tumors had genome regions for which the aCGH ratio was clearly different from that corresponding to an integer copy number. This probably reflected intratumor heterogeneity in the DNA copy numbers, that is, the existence of subpopulations with copy number changes that are not common for all tumor cells in the sample. The common aberrations can thus be considered homogeneous. Lymphomas and cervical cancers with heterogeneous DNA regions had ratio levels that fell in between, and were significantly different from, those corresponding to integer values (Figure 7). The actual ratio level reflected the proportion of cells with that aberration (Equation 1).

Nineteen (20%) lymphomas and 44 (50%) cervical cancers had one or more heterogeneous DNA regions with copy numbers 1&2, 2&3, or 3&4 (Additional data files 8 and 9). Reliable detection of heterogeneity required tumor cell fractions above 24% (Additional data file 10) and 5 out of 93 cervical cancers were therefore excluded from this analysis. Lymphoma L309/89 (Figure 7) had previously been identified as heterogeneous by FISH, showing one population with three and another with four copies of *MYC *and centromeres 8 and 17 [20]. Moreover, several of the heterogeneous aberrations in the cervical cancers, such as loss on chromosome 4 and X and gain on 11q and 17 in C005/01, loss on 6q and gain of 11q in C006/01, and loss on 4 in C023/01, were similar to those detected earlier by conventional CGH [14]. The previous study was, however, based on a different set of biopsies, which probably explains the lack of consistency for some of the tumors.

**Figure 8:**
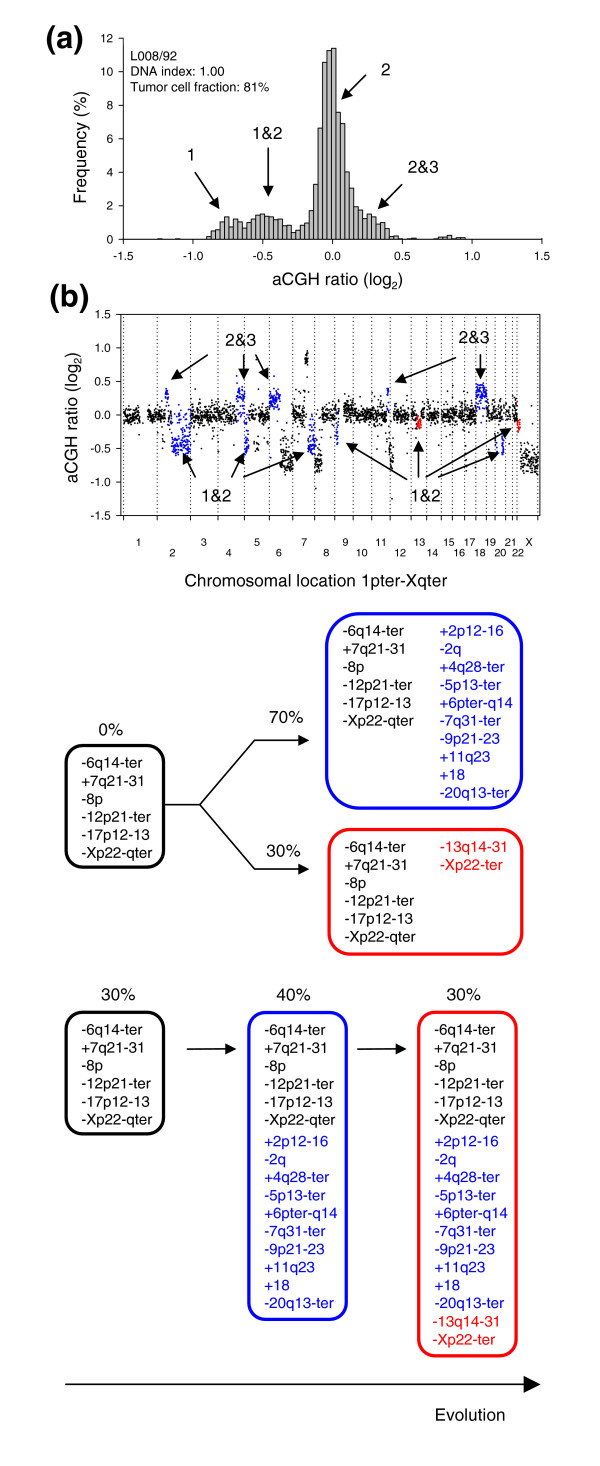
Evolutionary sequences of subpopulations in heterogeneous tumors. **(a) **Frequency histogram (% array probes) of aCGH ratios in a heterogeneous lymphoma is shown, including data for the entire genome. **(b) **The aCGH ratios are plotted against chromosomal location. The heterogeneous regions on chromosomes 2q, 5p, 7q, 9p, 13q, 20q, and Xp with a DNA copy number of 1&2 and on chromosomes 2p, 4q, 6p, 11q, and 18 with a DNA copy number of 2&3 are shown in blue and red. The blue and red colors represent aberrations that are present in different fractions of the tumor cells; 70% and 30%, respectively. The heterogeneous aberrations are listed in Additional data file 8 except those with a copy number of 2&3, since the lack of 3 DNA copies in this tumor prevented statistical analysis to identify 2&3 heterogeneity. **(c) **Schematic diagram of two possible evolutionary sequences for the aberrations, one parallel and one serial sequence, are shown. The blue and red circles represent the blue and red aberrations in (b). The percentages indicate the fractions of tumor cells with the listed aberrations, as calculated by GeneCount, showing that the aberrations in blue and red are present in 70% and 30% of the tumor cells, respectively.

In a few of the heterogeneous tumors, two different ratio levels were identified between one and two copies (Figure 8 and Additional data file 11). Thus, it appeared that the corresponding aberrations were present in different fractions of the tumor cell population. Lymphoma L008/92 had two intermediate ratio levels between one and two copies, corresponding to 70% and 30% of the tumor cells (Figure 8b, blue and red ratios, respectively), leading to the possible tumor evolutionary schemes depicted in Figure 8c. As the sum of the two fractions did not exceed 100%, the heterogeneous aberrations may be found in non-overlapping subpopulations of the tumor, where the subpopulations have evolved differently from a predicted common population containing the homogenous aberrations (parallel sequence). A serial sequence, where the populations have evolved in a linear manner from a common population, was also possible. In C024/01, however, the heterogeneous ratio levels corresponded to 78% and 44% of the tumor cells, and a serial sequence was the only one suggested (panel C in Additional data file 11).

## Discussion

We have shown that GeneCount is a reliable method for genome-wide calculation of DNA copy numbers in clinical tumor samples. Such data are biologically interesting in themselves but may also lead to improved prediction of treatment outcome and aid in the identification of novel tumor suppressors and oncogenes. We applied the method to lymphomas, for which accurate measures of tumor cell fraction and DNA copy numbers have been obtained by other techniques that could be compared with the GeneCount results. We further used the method on cervical cancers, for which tumor cell fractions representative of the aCGH data are more difficult to achieve by a separate technique. The GeneCount model is simple, due to the use of normal cells with two DNA copies throughout the genome as a reference sample. Moreover, the estimated copy numbers are restricted to positive integers, increasing the robustness of the method. A requirement for achieving the absolute quantification format is the use of pre-determined tumor ploidies, whereas the tumor cell fractions, if not known, and experimental bias can be estimated from the aCGH data.

The experimental bias is mainly caused by signals from unsuppressed repetitive sequences and nonspecific hybridization [2]. The bias influences the test and reference sample equally and independently of the DNA copy number, since it is generated by sequences distributed throughout the genome. The bias could, therefore, be summed up in an array specific factor, *q*, representing the dynamics of the log-transformed ratios. Mohapatra *et al*. [19] included the bias as a constant factor affecting the absolute, rather than the log-transformed, ratios in their model for pure tumor cells. Our approach seems justified because the noise (width) of the log-transformed ratios was independent of the ratios and, therefore, of the DNA copy numbers (Additional data file 6). We allowed for a small variation in *q *when calculating the tumor cell fraction to account for minor differences in the bias across the tumors. This *q*-value, optimized for each tumor, was highly similar to the mean *q *determined from control experiments, indicating that the bias was stable across experiments. Moreover, the discrepancies between the GeneCount and FISH results were related to the specific genetic aberration involved and, therefore, to the breakpoint detection algorithm, rather than to possible uncertainties in *q*. Recent developments in array CGH technology, utilizing oligonucleotides rather than bacterial artificial chromosome (BAC) clones, led to improved ratio dynamics and reduction in the experimental bias due to less repetitive sequences [28]. Ongoing work in our laboratory shows that by using oligoarrays, GeneCount can be applied with a *q *value close to 1.

Inclusion of the tumor cell fraction is a prerequisite for the calculation of absolute DNA copy numbers in clinical tumor samples. The lymphoma data were based on single cell suspensions made from the entire lymph nodes. A tumor cell fraction representative of the lymph node could, therefore, be determined with high accuracy by a separate technique like flow cytometry. In solid tumors such as cervical cancers, the normal cells consist of stroma, which is highly heterogeneously distributed within the tissue, and immune cells, which infiltrate the tumor parenchyma. A measure of the tumor cell fraction achieved by, for example, histological examination, which is based on a part of the sample different from that used for the aCGH experiment and/or fails to quantify the proportion of immune cells accurately, is, therefore, not precise enough for the calculation of DNA copy numbers. Histology data may, however, be useful for preselecting tumor enriched samples for the aCGH analysis. Fairly stable estimates of the tumor cell fraction, consistent with the values measured by flow cytometry, were achieved by the use of GeneCount. The estimates led to DNA copy numbers in agreement with the FISH data, suggesting that the accuracy of the tumor cell fractions was sufficient for reliable data analysis. Selection of appropriate ratio levels for the estimation was crucial for achieving this accuracy. We required that the tumors had at least two aberrations with different copy numbers and with more than ten array probes each to reduce errors caused by poorly defined ratio levels and breakpoints. Moreover, only ratio levels deviating more than 0.15 (log_2 _scale) from the baseline were selected, implying that tumor fractions higher than 24% (diploid) and 36% (tetraploid) were needed when copy numbers were changed to 3 or 5 copies, respectively (Additional data file 12).

The few discrepancies between the GeneCount and FISH data were not related to our model, but rather to the ability of the statistical methods to detect some of the aberrations. Hence, the consistency between the GeneCount and FISH results was similar to the reliability of GLAD in detecting breakpoints in simulated data [9]. The highest accuracy of the GeneCount results was obtained for well defined aberrant regions containing at least three array probes. In these cases a ratio level representative of the corresponding copy number was achieved and the probability of detecting the aberration was high. The increased uncertainty in the results of narrow aberrations implies that they should be confirmed by a separate technique like FISH. Moreover, to ensure sufficient ratio dynamics and, therefore, a high probability of breakpoint detection, a tumor cell fraction higher than a certain value, which depends on the experimental noise and tumor ploidy, is needed. With the noise of our experiments (Additional data file 6), a tumor cell fraction above 23% in diploid, and somewhat higher in hyperdiploid cases, enabled separation of an aberration with more than three array probes (Additional data file 13). This fraction also enabled detection of heterogeneous DNA copy numbers involving more than ten array probes (Additional data file 10). In experiments with more noise, caused by, for example, poor DNA quality, higher tumor cell fractions are required. In comparison, at least 50% tumor cells is suggested for optimal detection of gains and losses by conventional CGH [29].

The DNA copy number of genes involved in translocations cannot be directly assessed by FISH when a probe covering the breakage region is used, because signals from both the original chromosomes are detected in the translocated derivatives. Correction of the probe signal to achieve the true copy number requires knowledge of the breakpoint and genes involved in the translocation. Reliable FISH analysis in solid tumors, where the translocations are not well identified and may occur throughout the genome [30] is, therefore, particularly challenging. By aCGH, the probe signal is measured independently of the actual genome organization of the DNA covered by the probe. Hence, in the case of balanced translocations, a correct result will be obtained even if the probe covers the breakpoint. If the probe is located at the start or end of an amplified or deleted region (unbalanced translocation), the aCGH ratios of the adjacent probes ensure that the correct copy number is calculated. Our model therefore provides a novel method for assessment of copy numbers both in balanced and unbalanced translocated regions and without knowing that the translocation exists.

Current methods for analysis of aCGH data generally score genetic gains and losses based on ratio levels [31-36]. The breakpoints in individual tumors can be detected with high accuracy by use of statistical algorithms like GLAD and CGH-Explorer. However, the existing downstream analyses, using common ratio levels for scoring aberrations across tumors, fail to identify gains and losses in cases of high ploidy and normal cell content. By the use of GeneCount, the ratio levels are replaced with the absolute copy numbers relative to the total DNA content as measures of gene dosage, which can be compared across tumors regardless of ploidy and normal cell content. Hence, copy number changes that were not detected by analysis based on ratio levels, but showed significant correlation with gene expression, were found in cervical cancers, suggesting that improved results were achieved. Moreover, many patients with poor outcome that had *MRPS23 *gain by GeneCount had no gain based on ratio levels. In the latter case, the gain was masked by high content of normal cells or high ploidy, showing that GeneCount is more sensitive in detecting patients with genetic aberrations. The finding further demonstrates that GeneCount applies well to solid tumors for which the tumor cell fraction is generally unknown and must be estimated by the method. Advances in current statistical analysis methods may utilize adjustable ratio levels for scoring gains and losses, optimizing the cut-off ratios for each tumor based on a mathematical evaluation of the ratio dynamics. Such methods may account for varying ploidy and normal cell content across diploid, triploid, and tetraploid tumors. However, the strategy is not useful for tumors with an intermediate ploidy like 1.25 (Figure 1c). In contrast, the absolute DNA copy number relative to the total DNA content, or gene dosage, is comparable also across such tumors.

We also showed that GeneCount can provide genome-wide and high resolution information of intratumor heterogeneity in the DNA copy numbers. Such heterogeneity has previously been detected only on a single gene basis by FISH or at low resolution by conventional CGH analyses [14,15,20,37,38], probably reflecting a high genomic instability [39]. Detection of heterogeneity involving two DNA copies by the use of FISH is challenging, since the heterogeneous tumor population is difficult to distinguish from normal cells. The probability to detect heterogeneity with GeneCount depends on the fraction of tumor cells with the heterogeneous aberration. Obviously, the probability is largest at a fraction of 50%, but fractions higher than 70% and lower than 30% were also identified. Heterogeneity in low copy numbers, like 1&2 and 2&3, are more easily detected, since the separation between the log-transformed ratio levels are larger. At higher copy numbers, the possibility to detect heterogeneity decreases, depending on the ploidy and normal tissue content. However, we also identified heterogeneous regions with copy number 3&4 in several tumors and 4&5 in one tumor. Finally, the probability to detect heterogeneity also depends on the proportion of the genome that is affected. In our data severe heterogeneity affecting up to 40% of the genome could be analyzed with GeneCount (C002/01; Additional data file 9). With an increasingly larger part of the genome affected, difficulties in finding breakpoints and even homogeneous aberrations eventually occur, leading to unreliable results regardless of analysis method.

The heterogeneity data led to insight into the evolutionary sequence of the copy number changes. The homogeneous aberrations had probably occurred prior to the heterogeneous ones [14]. Moreover, in cases where the heterogeneous aberrations appeared to be present in different fractions of the tumor cell population, these aberrations could be ordered chronologically in a serial and/or parallel sequence. It was not always possible to identify the correct sequence among the proposed ones, as could be done by comparing data for several biopsies from the same tumor [14]. However, identification of the heterogeneous as well as the homogeneous aberrations suggests a further possible investigation of the exact combination of aberrations in each subpopulation, employing, for example, triple-color FISH with one probe for a homogeneous aberrant region and two for the heterogeneous ones.

In the heterogeneity analysis we assumed that the ploidy was the same for all subpopulations of malignant cells. This assumption was justified because no cases were observed with two aneuploid populations by flow cytometry. A possible difference in the ploidy of two aneuploid populations within a tumor was therefore probably smaller than 10%, leading to less than 10% uncertainty in the copy numbers calculated by GeneCount (Equation 4; data not shown). The same uncertainty also applied to near diploid and heterogeneous cervical cancers. These tumors often showed a broad G_1 _peak with a CV up to 10% by flow cytometry, probably reflecting the existence of several subpopulations with ploidy within the range of 1.0-1.1. Moreover, few or no light chain positive cells were observed in the diploid population of the aneuploid lymphomas, suggesting that the diploid population contained primarily normal cells. It is possible, however, that the diploid population of the aneuploid cervical cancers contained malignant cells, as we have previously shown for aneuploid colorectal cancers [40]. This might have led to larger uncertainties in the heterogeneous copy numbers due to the use of an erroneous DNA index of the diploid population. The data of such tumors can be improved by sorting the diploid and aneuploid fractions by flow cytometry [40] for separate aCGH and GeneCount analyses.

## Conclusion

GeneCount provides reliable DNA copy numbers, both when based on the tumor cell fractions determined by flow cytometry and those estimated by the method. Accurate data are also achieved in translocated chromosomal regions, as demonstrated for the t(14;18) translocation involving *BCL2*. Our method is the only one to provide genome-wide information of absolute DNA copy numbers. Moreover, the method represents a significant improvement compared to existing methods in the study of gene dosages and intratumor copy number heterogeneities. The robustness of GeneCount implies that the method can be utilized widely in the genomic exploration of both hematopoietic and solid tumors, addressing DNA copy number aspects in a reliable manner, regardless of possible translocations. This may lead to improved assays for disease classification and outcome prediction and aid the identification of efficient targets for new cancer therapies.

## Materials and methods

### Tumor samples, DNA index, and tumor cell fraction

Samples from 94 patients with B-cell non-Hodgkin's lymphoma and 99 patients with squamous cell carcinoma of the uterine cervix were analyzed. We used fresh frozen lymphoma cell suspensions for which the tumor subtype, stage, patient treatment, and follow-up have been presented previously [25]. The cervical cancers were of FIGO (Fédération Internationale des Gynaecologistes et Obstetristes) stage 1b-4b, treated with radiotherapy. Tumor biopsies taken before the start of treatment were used.

The *DI *of the lymphomas and cervical cancers and the tumor cell fraction of the lymphomas were determined by use of flow cytometry, and most of these data have been published earlier [14,23,25]. The lymphoma cells were labeled with phycoerythrin-labeled antibodies to the tumor characteristic light chains for identifying the tumor cells and Hoechst 33258 for assessment of DNA content. The *DI *was determined from the G_1 _peak position of the light chain positive cells relative to the light chain negative cells. Tumor cell fraction was determined as the fraction of light chain positive cells. The *DI *of the cervical cancers was assessed by preparing clean nuclei, stained with propidium iodide, using the detergent-trypsin method [41]. Cells from a diploid cell line were used as an internal reference. Samples showing two distinct G_1 _peaks in the DNA histogram were classified as aneuploid, and the *DI *was determined from the position of the G_1 _peak of the aneuploid cells relative to the corresponding peak of the diploid cells. Samples with a single G_1 _peak were classified as near diploid. An estimate of the tumor cell fraction was achieved for each cervical cancer sample by histological examination of hematoxylin and eosin stained sections derived from the middle part of the biopsies. These values were used to compare with the tumor cell fractions estimated by GeneCount.

### Array CGH

Genomic array slides produced by the Microarray Facility at the Norwegian Radium Hospital were used [42]. The arrays contained 4,549 unique genomic clones of BACs and P1 artificial chromosomes (PACs) (Wellcome Trust Sanger Institute, Cambridge, UK) that covered the whole genome with a resolution of approximately 1 Mb. The 1 Mb clone collection was supplemented with tiling path probes between 1q12 and 1q25, using overlapping BACs and PACs. The clones were from the RPCI-11 (BAC) and the RPC1-1, -3, -4, and -5 (PAC) libraries. Each clone was printed in 4-8 array spots. The genes covered by the clones were found from Ensembl [43].

Genomic DNA was isolated from the lymphoma cell suspensions and cervical cancer biopsies according to a standard protocol, including proteinase K, phenol, chloroform, and isoamylalcohol [44]. DNA (1 μg) was digested overnight, using *Dpn*II endonuclease (New England Biolabs, Beverly, MA, USA), and purified using the QIAquick PCR Purification Kit (Qiagen, Valencia, CA, USA). Digested and purified DNA and normal reference DNA (0.5 μg each) were labeled by a random primer reaction (BioPrime DNA Labeling System, Invitrogen, Carlsbad, CA, USA) with Cy3-dCTP and Cy5-dCTP (Perkin-Elmer Life Sciences, Foster City, CA, USA), respectively, and co-hybridized to the array slides [42]. Scanning and image analysis were performed by use of an Agilent scanner (Agilent Technologies Inc., Palo Alto, CA, USA) and the GenePix 6.0 image analysis software (Axon Instruments Inc., Union City, CA, USA). The microarray management and preprocessing software BASE [26] was used for spot filtering and ratio normalization. The mean value of the 4-8 spots of each genomic clone was used, provided that the standard deviation was less than 0.2. Lowess normalization was performed so that the mean log-transformed ratio of all clones was equal to 0. The GLAD and CGH-Explorer algorithms were used for ratio smoothing and breakpoint detection [9,11]. Default values of 8 (GLAD) and 1.5 (CGH-Explorer) for the statistical penalty, λ, were used. The smoothed ratios were inputs to GeneCount.

### Principle of GeneCount

For a heterogeneous test sample consisting of several cell populations, like normal cells and distinct populations of malignant cells, the DNA of each cell population contributes to the aCGH ratio. Ideally (that is, in cases of no experimental bias), the normalized ratio of each array probe is given by:

(1) $$R_{ideal}=\frac{\sum_{i=1}^{n}\frac{N_{i}}{2\cdot DI_{i}}\cdot F_{i}\cdot DI_{i}}{\sum_{i=1}^{n}F_{i}\cdot DI_{i}}$$

where *R*_ideal _is the aCGH ratio of a sample with *n *cell populations, and *N*_*i*_, *DI*_*i*_, and *F*_*i *_are the DNA copy number, DNA index, and tissue fraction of cell population *i*, respectively. We assume that: the reference sample is normal DNA with a copy number of 2 throughout the genome, except for the X and Y chromosomes in males; sex-matched hybridizations are performed; and *DI *is given relative to the DNA content of normal cells.

In cases of a homogeneous sample with a single cell population, for example, a cancer cell line, Equation 1 is reduced to:

(2) $$R_{ideal}=\frac{N}{2\cdot DI}$$

In clinical samples with two cell populations, that is, malignant and normal cells, the ratio is given by:

(3) $$R_{ideal}=\frac{N_{T}}{2\cdot DI_{T}}\cdot \frac{F_{T}\cdot DI_{T}}{F_{T}\cdot DI_{T}+(1-F_{T})}+\frac{1-F_{T}}{F_{T}\cdot DI_{T}+(1-F_{T})}$$

where *N*_*T*_, *DI*_*T*_, and *F*_*T *_are the DNA copy number, DNA index, and fraction of malignant cells in the sample, respectively. 1 - *F*_*T *_represents the fraction of normal cells, which have a *DI *of 1 and DNA copy number (*N*) of 2.

It was clear from experiments where normal male DNA was hybridized against female DNA that the ratio dynamics were somewhat reduced (Figure 1e). A dynamic factor, *q*, was included in Equation 3 to compensate for this effect. Since the experimental noise was independent of the logarithm of the ratio (Additional data file 6), Equation 3 was rewritten to account for the reduced dynamic in the following way:

(4) $$Log_{2}(R)=q\cdot Log_{2}\left(\frac{N_{T}}{2\cdot DI_{T}}\cdot \frac{F_{T}\cdot DI_{T}}{F_{T}\cdot DI_{T}+(1-F_{T})}+\frac{1-F_{T}}{F_{T}\cdot DI_{T}+(1-F_{T})}\right)$$

The dynamic factor represents the systematic, non-random reduction in the log-transformed ratios caused by the experimental bias and has a value between 0 and 1, where the latter value occurs in the ideal situation without any reduction in the ratio dynamics. The factor is a characteristic of the array slide series and the laboratory protocol and was determined from the ratio of the X chromosome in a control experiment hybridizing male versus female normal DNA (Figure 1e). Equation 4 was used in GeneCount to calculate *F*_*T *_and *N*_*T *_from the ratio profile of the sample.

Intratumor heterogeneity in the DNA copy numbers, that is, the cases of several populations of malignant cells in addition to the normal cells, was identified by selecting the tumors for which one or more of the aCGH ratio levels were different from that corresponding to an integer value by visual inspection. The ratio distributions of the potential heterogeneous regions were compared to the distributions of the adjacent homogeneous aberrations by ANOVA analysis, and a *P*-value of 0.05 was required to classify the aberration as heterogeneous. The fraction of tumor cells with a heterogeneous aberration was calculated, employing the more general Equation 1. The *DI *was assumed to be the same for all subpopulations of malignant cells.

### Implementation of GeneCount in BASE

We used BASE as a platform for GeneCount and linked the algorithm to the output of the GLAD and CGH-Explorer packages, which were implemented in our BASE version. The method can also be developed as a separate program or integrated in other aCGH analysis packages. The algorithm consists of three major steps: data input for all samples; estimation of tumor cell fraction in the cases when this parameter is unknown; and estimation of DNA copy number for each array probe (panel A in Additional data file 1). The smoothed aCGH ratios served as input, together with the *DI*, the *q*-value from control experiments with its lower and upper limits (*q*_min_, *q*_max_) and, if available, the tumor cell fraction.

In cases of unknown tumor cell fraction, this value was estimated in a simulation procedure based on two selected ratio levels, using the tumor cell fraction and DNA copy numbers as independent and *q *as dependent variables. The copy numbers and tumor cell fraction were increased in steps of 1 and 0.01, respectively, and the corresponding *q*-value was calculated (panel B in Additional data file 1). To ensure high accuracy in the estimated fractions, it was required that the absolute value of the selected ratio levels was larger than 0.15. This implied that samples with a tumor cell fraction lower than 24% in diploid and 36% in tetraploid tumors could not be analyzed when only aberrations involving one copy number change existed (Additional data file 12). Moreover, a minimum absolute difference of 0.2 - that is, approximately two times the standard deviation of the log-transformed ratio levels (Additional data file 6) - between the two selected ratio levels was needed. To further increase the reliability of the estimation, only ratio levels with more than ten probes were selected. We optimized *q *for each tumor by allowing the value to vary within the limited range of *q*_min _to *q*_max_, typically *q *± 10%, leading to fairly stable estimates of the tumor cell fraction. The mean tumor cell fraction based on these estimates and the corresponding mean *q*-value was used in Equation 4 to estimate the DNA copy numbers of the tumor. In cases of known tumor cell fraction, this fraction and *q *from control experiments were used in Equation 4. The source code of the module is provided by communication to the authors. A demo version of GeneCount in BASE is also available [45].

### Fluorescence *in situ *hybridization

GeneCount estimates for the lymphomas were compared with direct assessments of gene copy numbers by use of FISH. All FISH analyses have been published previously [20-25]. Dual-color FISH was applied to all 94 tumors. We used spectrum orange labeled locus-specific propidium iodide DNA probes for genes commonly aberrant in lymphomas (*CCND3*, *BMP6*, *PIM1*, *MYC*, *CDKN2A*, *RB1*, *TP53*, *PMAIP1*, and *MALT1*) and spectrum green labeled centromer probes (centromere 1, 6, 8, 17, and 18) (Vysis Inc., Downers Grove, IL, USA) for assessing the quality of the experiment. For exploring DNA copy number calculations in translocated chromosomal regions, *BCL2*, which is frequently involved in the translocation t(14;18)(q32;q21) in lymphomas, was considered. A dual-color translocation probe involving *BCL2 *and covering the breakpoint region was used (LSI *IGH *Spectrum Green/LSI *BCL2 *Spectrum Orange, Vysis Inc.). Due to splitting of the probe signal in cases of translocation, erroneous high *BCL2 *copy numbers were derived directly with this probe. The *BCL2 *copy number was therefore corrected based on the signals from the *IGH *and centromere 18 probes, as described [22].

### Gene expression microarrays

Gene expressions were determined by microarray analysis of 89 of the cervical cancers and related to the GeneCount estimates. We used array slides produced at the Microarray Facility at the Norwegian Radium Hospital, containing 15,000 cDNA clones. The data from 48 of the patients, with a detailed description of the experimental procedures, have been presented [27]. Cy3- and Cy5-labeled cDNA was synthesized from total RNA by anchored oligo(dT)-primed reverse transcription and co-hybridized with a reference sample (Universal Human Reference RNA, Stratagene, La Jolla, CA, USA) to the array slides overnight at 65°C. Scanning and image analysis were performed with an Agilent scanner and the GenePix 4.1 image analysis software, respectively. Data preprocessing, including correction of saturated intensities, filtering of weak and bad spots, and lowess normalization, was performed in BASE. All hybridizations were performed twice in a dye-swap design, and the average expression ratio based on the two experiments was used in the further analyses.

### ArrayExpress accession

The array CGH raw data have been deposited to the ArrayExpress repository (E-TABM-398, E-TABM-399).

## Abbreviations

aCGH, array comparative genomic hybridization; BAC, bacterial artificial chromosome; BASE, Bioarray Software Environment; DI, DNA index; FISH, fluorescence *in situ *hybridization; PAC, P1 artificial chromosome.

## Authors' contributions

HL and TS conceived and designed the study and analyzed data. HL wrote the article, ML, RSB, DHS, EG, and OTB carried out the aCGH, FISH, and flow cytometry experiments and participated in data analysis, LAMZ and OM contributed to the aCGH experiments, MJ and EH contributed to the implementation of GeneCount in BASE, GBK provided clinical samples and data, and TS helped to draft the manuscript. All authors read and approved the final manuscript.

## Additional data files

The following additional data are available with the online version of this paper. Additional data file 1 is a figure showing the calculation steps in GeneCount. Additional data file 2 is a figure showing an example of FISH probe locations. Additional data file 3 is a figure comparing FISH DNA copy numbers and smoothed aCGH ratio levels in non-Hodgkin's lymphomas. Additional data file 4 is a figure illustrating discrepancies between GeneCount and FISH DNA copy numbers. Additional data file 5 is a figure comparing tumor cell fractions derived by histological examination and by GeneCount estimation in cervical cancers. Additional data file 6 is a figure showing the standard deviation (noise) of the log-transformed aCGH ratios. Additional data file 7 is a figure comparing results from ratio level and GeneCount analyses in cervical cancers. Additional data file 8 is a table listing regions with DNA copy number heterogeneity in non-Hodgkin's lymphomas. Additional data file 9 is a table listing regions with DNA copy number heterogeneity in cervical cancers. Additional data file 10 is a figure showing tumor cell fraction required for detection of heterogeneous copy number changes. Additional data file 11 is a figure illustrating analysis of the evolutionary sequence of subpopulations in heterogeneous tumors. Additional data file 12 is a figure showing the minimum tumor cell fraction that can be calculated in GeneCount. Additional data file 13 is a figure showing the tumor cell fraction required for detection of homogeneous copy number changes.

## Supplementary Material

Additional data file 1 Calculation steps in GeneCount. Click here for file

Additional data file 2 An example of FISH probe locations. Click here for file

Additional data file 3 Comparison of FISH DNA copy numbers and smoothed aCGH ratio levels in non-Hodgkin's lymphomas. Click here for file

Additional data file 4 Discrepancies between GeneCount and FISH DNA copy numbers. Click here for file

Additional data file 5 Comparison of tumor cell fractions derived by histological examination and by GeneCount estimation in cervical cancers. Click here for file

Additional data file 6 Standard deviation (noise) of the log-transformed aCGH ratios. Click here for file

Additional data file 7 Comparison of results from ratio level and GeneCount analyses in cervical cancers. Click here for file

Additional data file 8 Regions with DNA copy number heterogeneity in non-Hodgkin's lymphomas. Click here for file

Additional data file 9 Regions with DNA copy number heterogeneity in cervical cancers. Click here for file

Additional data file 10 Tumor cell fraction required for detection of heterogeneous copy number changes. Click here for file

Additional data file 11 Analysis of the evolutionary sequence of subpopulations in heterogeneous tumors. Click here for file

Additional data file 12 The minimum tumor cell fraction that can be calculated in GeneCount. Click here for file

Additional data file 13 The tumor cell fraction required for detection of homogeneous copy number changes. Click here for file
