# Supplementary material for: GeneCount: genome-wide calculation of absolute tumor DNA copy numbers from array comparative genomic hybridization data
Source: Genome Biol. 2008 May 23;9(5):R86. doi: 10.1186/gb-2008-9-5-r86 (PMC2441472; doi:10.1186/gb-2008-9-5-r86)
Supplement: Additional data file 1 — Calculation steps in GeneCount. [file gb-2008-9-5-r86-S1.pdf]

Additional data file 1

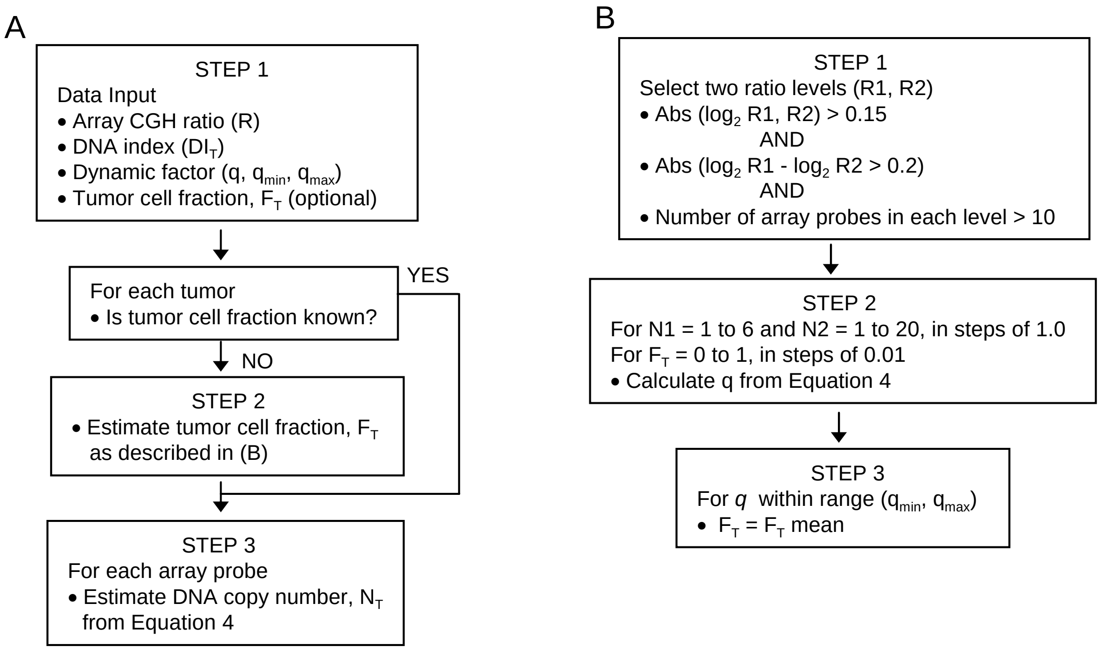

Calculation steps in GeneCount.
Float diagrams of the entire GeneCount procedure (A) and the estimation of tumor cell fraction (B) are shown. Data input in (A) includes the aCGH ratio levels, *R*, derived from statistical analysis tools, the DNA index (DI_T) of the tumor cells, the dynamic factor, *q*, of the experiment, and the tumor cell fraction, F_T. F_T can either be measured by a separate technique like flow cytometry, or estimated by the procedure in (B). In the former case, a fixed *q*-value, as determined from control experiments, is used. Otherwise, *q* is estimated for each tumor as indicated in (B), allowing for a deviation of typically 10% (range *q*min - *q*max) from the value determined in control experiments. To calculate tumor cell fraction, two ratio levels, *R*1 and *R*2, are selected, and the DNA copy numbers *N*1 and *N*2, corresponding to *R*1 and *R*2, respectively, are predicted in a stepwise manner by increasing *N*1 from 1 to 6 and *N*2 from 1 to 20 in steps of 1. *R*1, *R*2, and the incremental values of *N*1 and *N*2 are used in a simulation procedure based on Equation 4, allowing F_T to vary from 0 to 1 in steps of 0.01, and calculating the corresponding *q*. The F_T values obtained for *q* within the range *q*min – *q*max are used to calculate a mean F_T representative of the tumor. The mean F_T is further used as input to the algorithm in (A) to calculate the DNA copy numbers of all array probes. The criteria used to select *R*1 and *R*2 for estimation of tumor cell fraction are described in the Materials and methods section of the paper. The source code of the GeneCount module is available by communication to the authors.
