# Supplementary material for: GeneCount: genome-wide calculation of absolute tumor DNA copy numbers from array comparative genomic hybridization data
Source: Genome Biol. 2008 May 23;9(5):R86. doi: 10.1186/gb-2008-9-5-r86 (PMC2441472; doi:10.1186/gb-2008-9-5-r86)
Supplement: Additional data file 2 — An example of FISH probe locations. [file gb-2008-9-5-r86-S2.pdf]

Additional data file 2

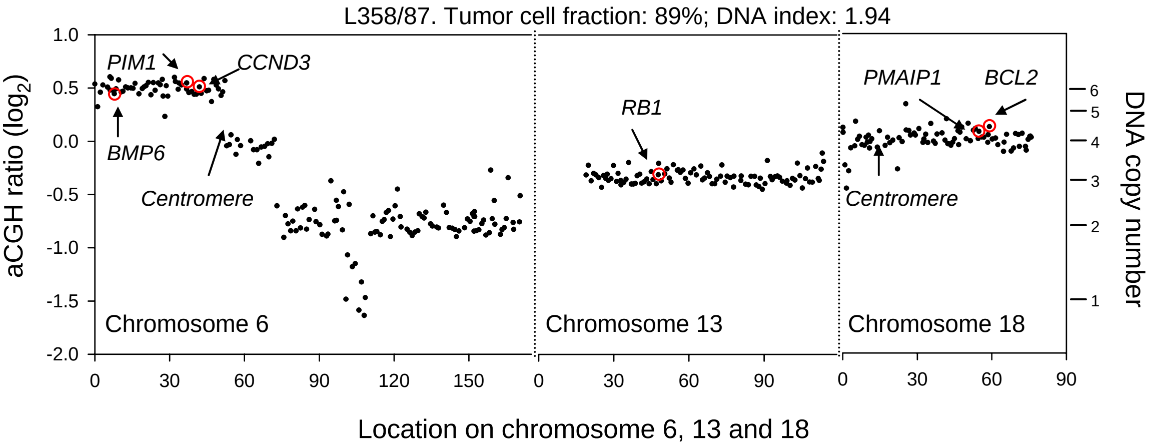

Example of FISH probe locations.

ACGH ratios are plotted against chromosomal location on chromosomes 6, 13, and 18 in a tetraploid lymphoma with a tumor cell fraction of 89%. FISH probes for *BMP6*, *PIM1*, *CCND3*, centromere 6, *RB1*, *PMAIP1*, *BCL2*, and centromere 18 were used in this particular tumor, and their location is marked. The copy numbers determined with FISH were 3 (*RB1*), 4 (*PMAIP1*, *BCL2*), and 6 (*BMP6*, *PIM1*, *CCND3*).
