# Supplementary material for: GeneCount: genome-wide calculation of absolute tumor DNA copy numbers from array comparative genomic hybridization data
Source: Genome Biol. 2008 May 23;9(5):R86. doi: 10.1186/gb-2008-9-5-r86 (PMC2441472; doi:10.1186/gb-2008-9-5-r86)
Supplement: Additional data file 3 — Comparison of FISH DNA copy numbers and smoothed aCGH ratio levels in non-Hodgkin's lymphomas. [file gb-2008-9-5-r86-S3.pdf]

Additional data file 3

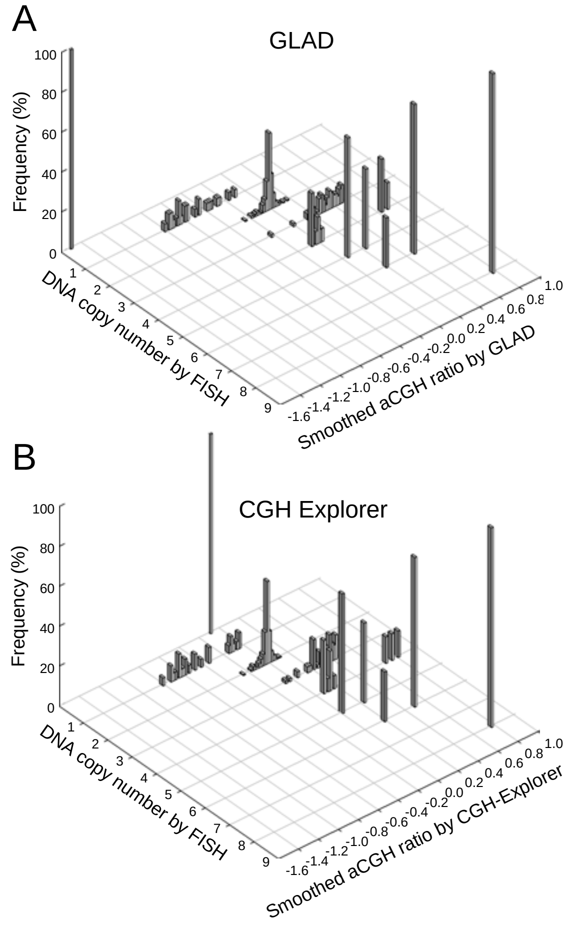

FISH DNA copy numbers in relation to smoothed aCGH ratio levels.
Smoothed aCGH ratio levels derived from GLAD (A) and CGH-Explorer (B) is plotted against the corresponding FISH results for 9 genes in 94 lymphomas. Frequency distributions are shown for each copy number, containing 1, 25, 246, 66, 15, 5, 4, and 1 values at a FISH copy number of 0, 1, 2, 3, 4, 5, 6, and 8, respectively.
