# Supplementary material for: GeneCount: genome-wide calculation of absolute tumor DNA copy numbers from array comparative genomic hybridization data
Source: Genome Biol. 2008 May 23;9(5):R86. doi: 10.1186/gb-2008-9-5-r86 (PMC2441472; doi:10.1186/gb-2008-9-5-r86)
Supplement: Additional data file 4 — Discrepancies between GeneCount and FISH DNA copy numbers. [file gb-2008-9-5-r86-S4.pdf]

Additional data file 4

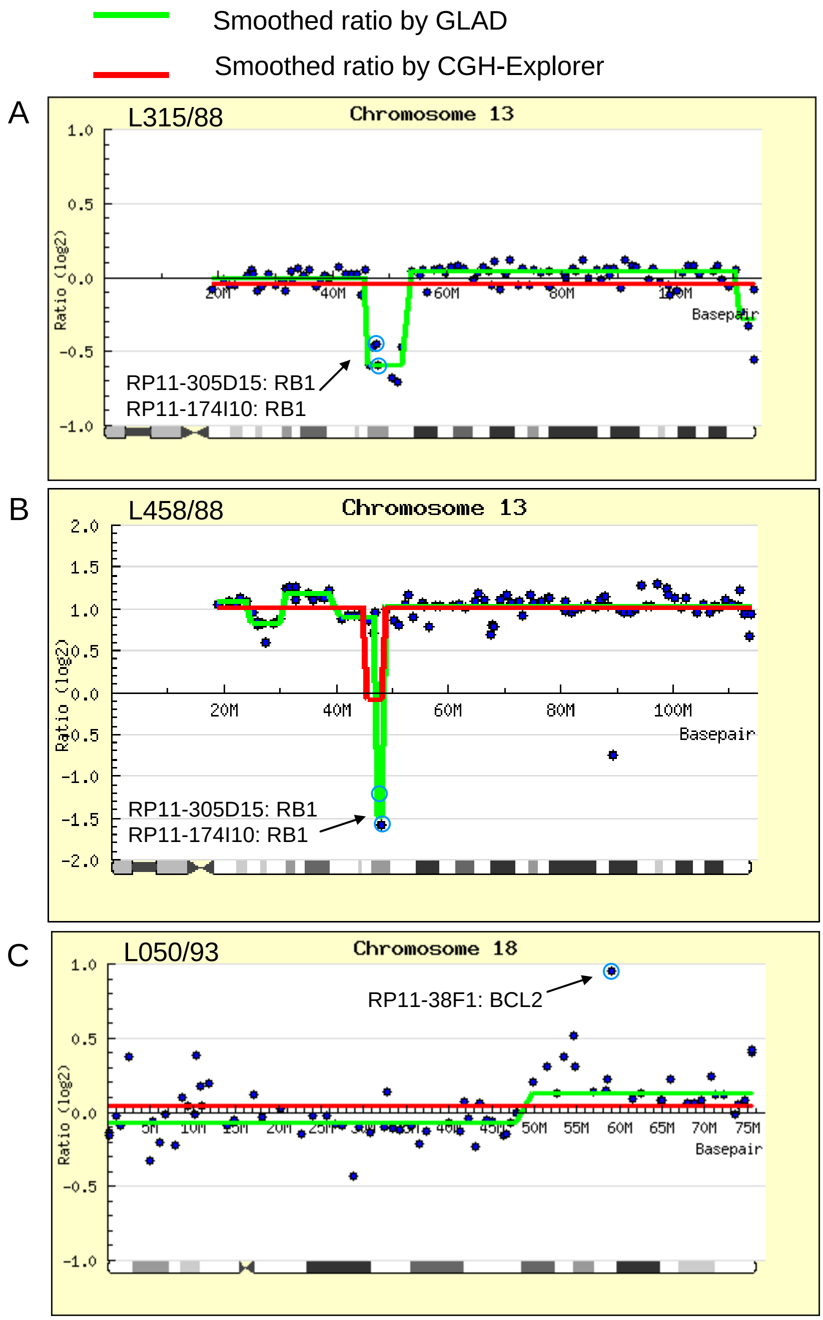

Discrepancies between GeneCount and FISH DNA copy numbers.
(A) shows a deletion involving *RB1* that was detected by GLAD but not by CGH-Explorer. The GeneCount estimate was 1.1 and 1.9 based on GLAD and CGH-Explorer, respectively. The *RB1* copy number determined by FISH was 1. (B) shows a homozygote deletion involving *RB1*, where the ratio level was inappropriately determined since the deleted region contained only two array probes. The GeneCount estimate was 0.5 and 1.9 based on GLAD and CGH-Explorer, respectively. The *RB1* copy number determined by FISH was zero. (C) shows a gain involving *BCL2*, which was not detected by GLAD or CGH-Explorer. The GeneCount estimate was 2.3 and 2.1 based on GLAD and CGH-Explorer, respectively. The *BCL2* copy number determined by FISH was 5. There was no t(14;18) translocation in this tumor.
