# Supplementary material for: GeneCount: genome-wide calculation of absolute tumor DNA copy numbers from array comparative genomic hybridization data
Source: Genome Biol. 2008 May 23;9(5):R86. doi: 10.1186/gb-2008-9-5-r86 (PMC2441472; doi:10.1186/gb-2008-9-5-r86)
Supplement: Additional data file 5 — Comparison of tumor cell fractions derived by histological examination and by GeneCount estimation in cervical cancers. [file gb-2008-9-5-r86-S5.pdf]

Additional data file 5

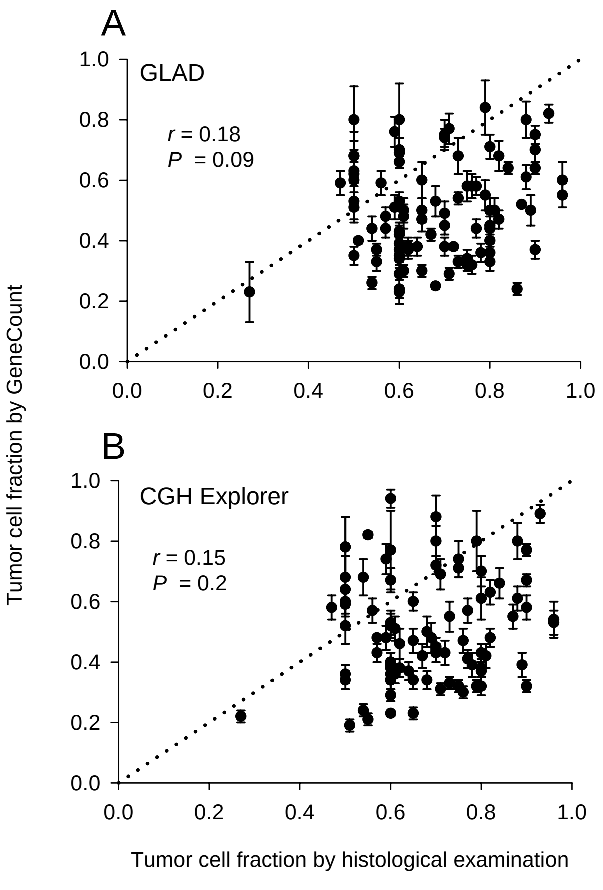

Histological examination and GeneCount estimation of tumor cell fraction.
Plots of tumor cell fraction of cervical cancer samples estimated by GeneCount versus tumor cell fraction measured by histological examination of tumor sections. Each point represents mean ± SD based on the values achieved for q within the range 0.7 – 0.9. The smoothed aCGH ratios from GLAD (A) and CGH-explorer (B), the q range 0.7 – 0.8, and a *DI* determined by flow cytometry were inputs to GeneCount. The calculations were based on 93 (A) and 89 (B) tumors, for which suitable ratio levels for the calculations existed. Correlation coefficients and P-values from Pearson product moment correlation analyses are indicated.
