# Supplementary material for: GeneCount: genome-wide calculation of absolute tumor DNA copy numbers from array comparative genomic hybridization data
Source: Genome Biol. 2008 May 23;9(5):R86. doi: 10.1186/gb-2008-9-5-r86 (PMC2441472; doi:10.1186/gb-2008-9-5-r86)
Supplement: Additional data file 6 — Standard deviation (noise) of the log-transformed aCGH ratios. [file gb-2008-9-5-r86-S6.pdf]

Additional data file 6

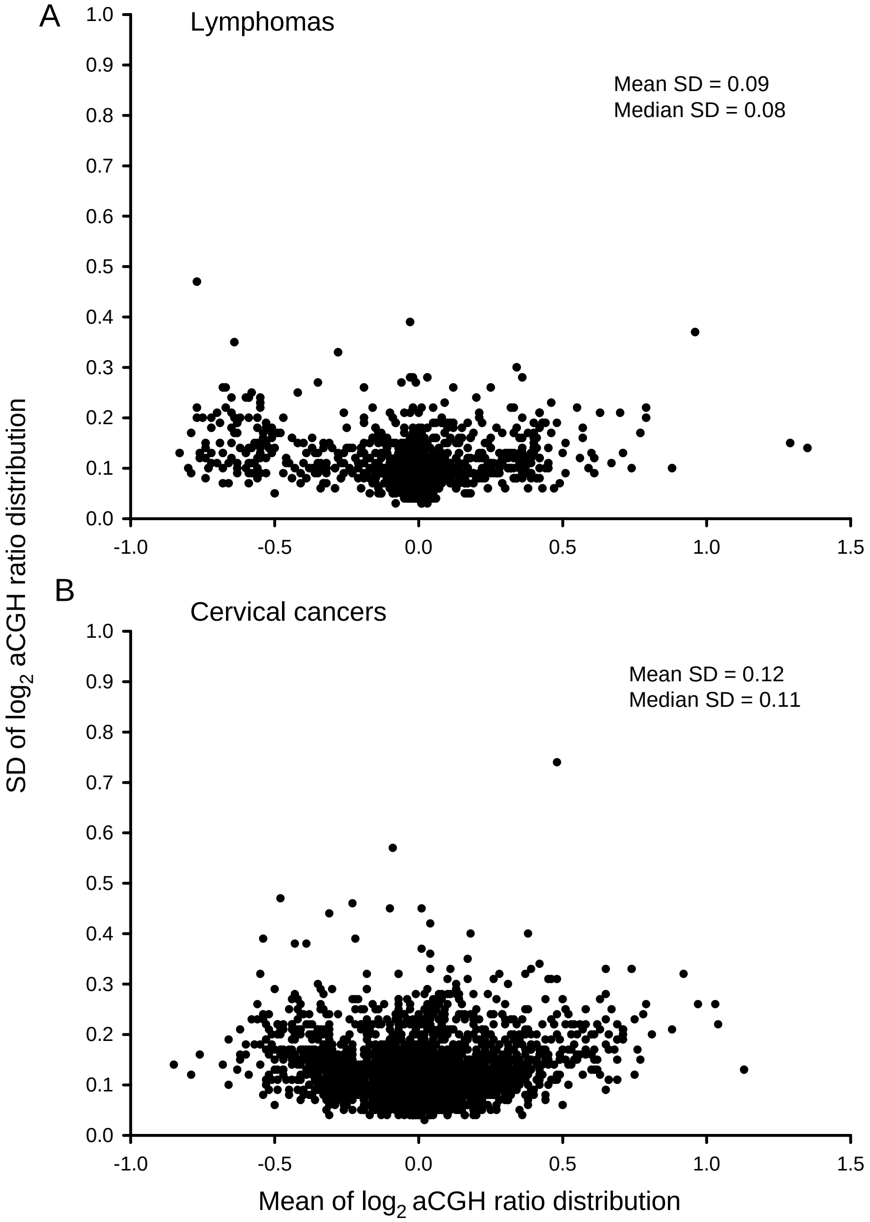

Standard deviation (noise) of log transformed aCGH ratios.
Standard deviation (SD) *versus* mean of the log-transformed ratio distributions achieved for 94 lymphomas (A) and 99 cervical cancers (B) is shown. The individual ratio levels determined by the breakpoint detection algorithm in GLAD were considered. The median and mean SD is indicated. Note that the SD was independent of the mean ratio and therefore of the DNA copy number.
