# Supplementary material for: GeneCount: genome-wide calculation of absolute tumor DNA copy numbers from array comparative genomic hybridization data
Source: Genome Biol. 2008 May 23;9(5):R86. doi: 10.1186/gb-2008-9-5-r86 (PMC2441472; doi:10.1186/gb-2008-9-5-r86)
Supplement: Additional data file 7 — Comparison of results from ratio level and GeneCount analyses in cervical cancers. [file gb-2008-9-5-r86-S7.pdf]

Additional data file 7

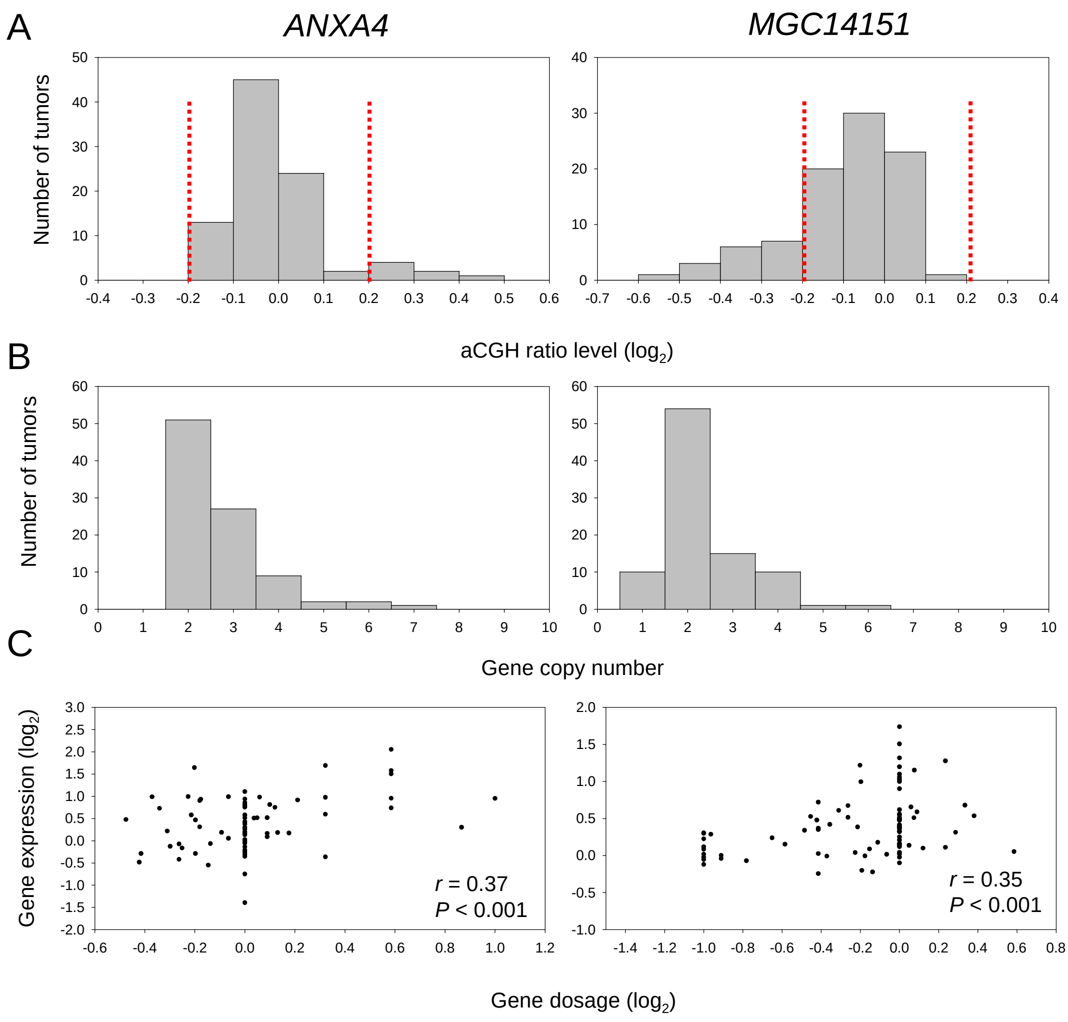

Ratio level and GeneCount analyses in cervical cancers.

In (A) frequency histogram (number of tumors) of smoothed aCGH ratios (GLAD) is shown for *ANXA4* (BAC clone ID RP11-304A15) (left panel) and *MGC14151* (RP11-144K9) (right panel). Stippled lines indicate the cut off ratios of ±0.2, identifying 7 (*ANXA4*) and 17 (*MGC14151*) tumors with genetic gain or loss. In (B) frequency histogram (number of tumors) of DNA copy number calculated by GeneCount is shown for *ANXA4* (left panel) and *MGC14151* (right panel). The GLAD ratio levels, the *DI* measured by flow cytometry, and the tumor cell fraction estimated by GeneCount were used in the calculation. Similar results were achieved based on the CGH-Explorer ratio levels. In (C) plot of gene expressions against gene dosage; *i.e.*, the *ANXA4* (left panel) and *MGC14151* (right panel) copy number divided by the total DNA content ($N/(2 \cdot DI)$), is shown. The gene dosage was changed; *i.e.*, higher than 0.2 or lower than -0.2, in 26 (*ANXA4*) and 40 (*MGC14151*) tumors; *i.e.*, in considerable more tumors than when only the ratio levels were used. Correlation coefficient and *P*-value from Pearson product moment correlation analysis are indicated. Panels A and B are based on 92 (ANXA4) and 91 (MGC14151) tumors, for which GeneCount data existed. Panel C is based on 85 of these tumors, for which both DNA copy numbers and expression were available.
