# Supplementary material for: GeneCount: genome-wide calculation of absolute tumor DNA copy numbers from array comparative genomic hybridization data
Source: Genome Biol. 2008 May 23;9(5):R86. doi: 10.1186/gb-2008-9-5-r86 (PMC2441472; doi:10.1186/gb-2008-9-5-r86)
Supplement: Additional data file 8 — Regions with DNA copy number heterogeneity in non-Hodgkin's lymphomas. [file gb-2008-9-5-r86-S8.pdf]

Additional data file 8

Table 1. DNA copy number heterogeneity in non-Hodgkin's lymphomas

Patient	DNA index	DNA region[a]	DNA copy number
L122/84	1.23	17q24-ter	1&2
L255/85	1.04	12p	1&2
L358/87	1.94	9pter-q21, 9q31-ter	3&4
L47/88	1.00	11q14-22	1&2
L154/88	0.95	4	1&2
L399/88	1.00	11q13-14, X (male)	1&2
		22	2&3
L064/89	1.26	17	2&3
L309/89	1.83	8, 9, 17q21-ter	3&4
L339/89	1.00	17q21-25	2&3
L034/90	1.16	X (male)	1&2
L472/90	1.16	X	2&3
L577/90	1.97	4	2&3
		11	3&4
		3	4&5
L382/91	1.02	13q32-ter	1&2
L383/91	0.97	11q22-23, 15q25-ter	1&2
L436/91	1.04	Xq26-ter (male)	1&2
L462/91	1.00	3pcen-14, 3p21-22, 3p24-ter, 15qcen-15, 17p11-ter	1&2
L008/92	1.00	2q, 5p13-ter, 7q31-ter, 9p21-23, 13q14-31, 20q13-ter, Xp22-ter	1&2
L037/92	1.00	19	1&2
L117/92	1.00	4	1&2

[a]The aCGH ratios of the heterogeneous DNA region were significantly different from those of the homogeneous regions in all cases, as verified from ANOVA analysis.
