# Supplementary material for: GeneCount: genome-wide calculation of absolute tumor DNA copy numbers from array comparative genomic hybridization data
Source: Genome Biol. 2008 May 23;9(5):R86. doi: 10.1186/gb-2008-9-5-r86 (PMC2441472; doi:10.1186/gb-2008-9-5-r86)
Supplement: Additional data file 9 — Regions with DNA copy number heterogeneity in cervical cancers. [file gb-2008-9-5-r86-S9.pdf]

Additional data file 9

Table 2. DNA copy number heterogeneity in cervical cancers

Patient	DNA index	DNA region[a]	DNA copy number
C002/01	1.00	1p34-ter, 10, 16, 17, 19, 20	1&2
		2pter-q33, 5p14-q14, 6, 7p11-ter, 8, 12pter-q23, X	2&3
C003/01	1.90	5pter-q12, 6, 11pter-q12, 12, 13q21-31, Xp	3&4
C004/01	1.00	17q23-ter, X	2&3
C005/01	1.70	1p33-ter, 4, 7q, Xpter-q27	2&3
		3q, 6q13-ter, 7p, 10pter-q24, 11pter-q22, 17, 18, 20	3&4
C006/01	1.30	6q24-ter	1&2
		1p32-ter, 11q12-13, 17q, 18, 19, 22q	2&3
C008/01	1.00	9q	2&3
C014/01	1.94	3p	3&4
C023/01	1.00	2q33-ter, 4, 8p	1&2
		1p32-qter, 3, 7q21-ter	2&3
C024/01	1.00	1p31-ter, 2q21-ter, 4p, 8p, 13qcen-34	1&2
		6p	2&3
C025/01	1.00	21qcen-21, X	1&2
		9	2&3
C028/01	1.00	X	1&2
		9	2&3
C041/01	1.34	8	3&4
C045/01	1.00	3p	1&2
C053/01	1.00	1p34-ter	1&2
C054/01	1.00	2q33-ter, 9qcen-22, Xq22-ter	1&2
		15	2&3
C078/02	1.70	5q, Xpter-q28	3&4
C092/02	1.00	3p, 8p12-ter, 17, 19, 20, 22	1&2
		1p34-ter, 2p13-ter, 8p12-q11, 8q13-ter, 13q, 18	2&3
C093/02	2.00	17pter-q25, 18	3&4
C101/02	1.50	2, 4, 20q11-ter	2&3
		1, 3p	3&4
C115/03	1.00	2p21-11, 17q21-ter, 18q21-ter	2&3
C120/03	1.00	22	1&2
		8, 18, X	2&3

C130/03	1.60	4, 5qcen-14, 7, 17pter-q21	2&3
		12q, Xq	3&4
C136/03	1.40	4p15-ter, 19pter-q13	1&2
		6p12-ter, 8q22-ter, X	2&3
C138/03	1.90	7p11-22, 12, 15	3&4
C141/03	1.10	5q23-ter, 20q12-ter	2&3
C142/03	1.00	3q24-ter, X	2&3
C144/03	1.50	3p, 19	2&3
		3q, 4q13-ter, 7, Xp22-q21	3&4
C152/03	1.00	3q	2&3
C162/04	2.16	10q, 12q, 18q, 21	3&4
C169/04	1.00	18q12-ter, 21qcen-21	1&2
C174/04	1.95	1p32-ter, 16, 19, 20, 22	3&4
C178/04	1.00	5q, 17p, 21	1&2
		1q, 13q22-ter, 16, 20	2&3
C180/04	1.92	11p, 18q11-21,	2&3
		3p, 7, 13q32-ter, 18q21-ter	3&4
C184/04	1.39	3p, 14, 17p, Xpter-q25	2&3
		1pter-q32, 8q21-ter	3&4
C188/04	1.00	2, 5q, 6pter-q25, 10, 11, 15, 18	1&2
		9q22-ter, 12p11-ter, 12q13-ter, 16	2&3
C189/04	1.50	4q, Xp11-ter	1&2
		17pter-q24	2&3
C190/04	1.00	3p, 4, 8, 13	1&2
C193/04	1.00	1pcen-31, 3p, 4p15-qter, 5, 6p21-q24, 8, 14qcen -21, 18	1&2
C194/04	1.00	5q, 9p, 11p15-q13, 18p12-ter, 22q13-ter	1&2
		16q, 19p, 20	2&3
C195/04	1.90	1p32-q12, 2p11-ter, 12q, 14, 15	3&4
C196/04	1.00	2q12-q33, 4p13-qter, 17p, X	1&2
C202/04	1.00	13q21-q32	1&2
C206/04	1.00	4, 13q21-q31	1&2
		1p31-ter, 19, 22	2&3
C215/04	1.00	11p, 13q21-ter	1&2
		1p31-ter	2&3

[a]The aCGH ratios of the heterogeneous DNA region were significantly different from those of the homogeneous regions in all cases, as verified from ANOVA analysis.
