# Supplementary material for: GeneCount: genome-wide calculation of absolute tumor DNA copy numbers from array comparative genomic hybridization data
Source: Genome Biol. 2008 May 23;9(5):R86. doi: 10.1186/gb-2008-9-5-r86 (PMC2441472; doi:10.1186/gb-2008-9-5-r86)
Supplement: Additional data file 10 — Tumor cell fraction required for detection of heterogeneous copy number changes. [file gb-2008-9-5-r86-S10.pdf]

Additional data file 10

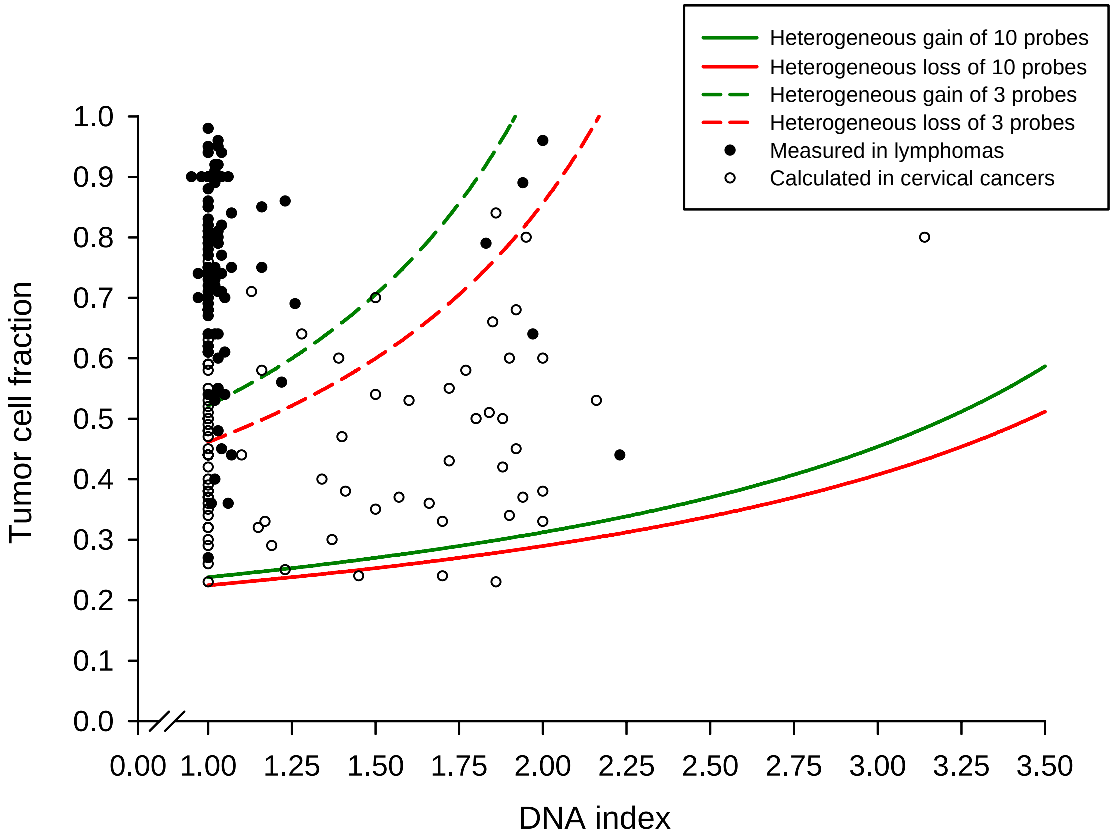

Tumor cell fraction required for detection of heterogeneous copy number changes.
The tumor fraction needed for statistically significant separation of a heterogeneous chromosomal region involving more than ten (solid lines) or three (stippled lines) array probes from a homogeneous region without aberration is shown as a function of the DNA index (*DI*). The curve for heterogeneous gain (copy number 2·*DI* + 0.5) and loss (copy number 2·*DI* - 0.5) is shown in green and red color, respectively. A Student t-test and a standard deviation of 0.1 for the log-transformed ratio distributions were used to estimate the curves. It was assumed that the heterogeneous gain and loss affected 50% of the tumor cells in the sample. Data showing the tumor cell fraction of 94 lymphomas (closed symbols) and 93 cervical cancers (open symbols), as determined by flow cytometry (lymphomas) and estimated by GeneCount from the GLAD ratio levels (cervical cancers) are included.
