# Supplementary material for: GeneCount: genome-wide calculation of absolute tumor DNA copy numbers from array comparative genomic hybridization data
Source: Genome Biol. 2008 May 23;9(5):R86. doi: 10.1186/gb-2008-9-5-r86 (PMC2441472; doi:10.1186/gb-2008-9-5-r86)
Supplement: Additional data file 11 — Analysis of the evolutionary sequence of subpopulations in heterogeneous tumors. [file gb-2008-9-5-r86-S11.pdf]

Additional data file 11

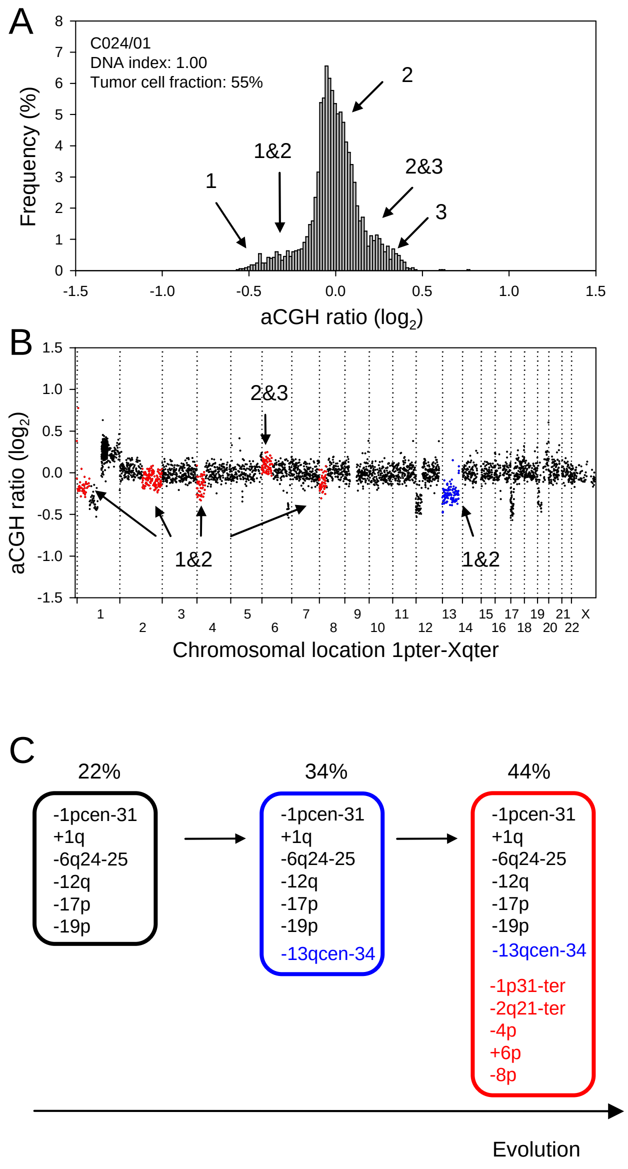

Evolutionary sequence of subpopulations in heterogeneous tumors.
In (A) frequency histogram (% array probes) of aCGH ratios in a heterogeneous cervical cancer is shown. Data of the entire genome are included. In (B) the aCGH ratios are plotted against chromosomal location. The heterogeneous regions on chromosomes 1p, 2q, 4p, 8p, and 13q with a DNA copy number of 1&2 and on 6p with a DNA copy number 2&3 are shown in blue and red. The blue and red colors represent aberrations of two different fractions of the tumor cells, 78% and 44%, respectively. The heterogeneous aberrations are listed in Additional Data File 9. In (C) schematic diagram of the possible evolutionary sequence for the aberrations are shown. The blue and red aberrations refer to the blue and red aberrations in (B). The percentages indicate the fractions of tumor cells with the listed aberrations, as calculated by GeneCount, showing that the aberrations in blue and red are present in 78% and 44% of the tumor cells, respectively.
