# Supplementary material for: GeneCount: genome-wide calculation of absolute tumor DNA copy numbers from array comparative genomic hybridization data
Source: Genome Biol. 2008 May 23;9(5):R86. doi: 10.1186/gb-2008-9-5-r86 (PMC2441472; doi:10.1186/gb-2008-9-5-r86)
Supplement: Additional data file 12 — The minimum tumor cell fraction that can be calculated in GeneCount. [file gb-2008-9-5-r86-S12.pdf]

Additional data file 12

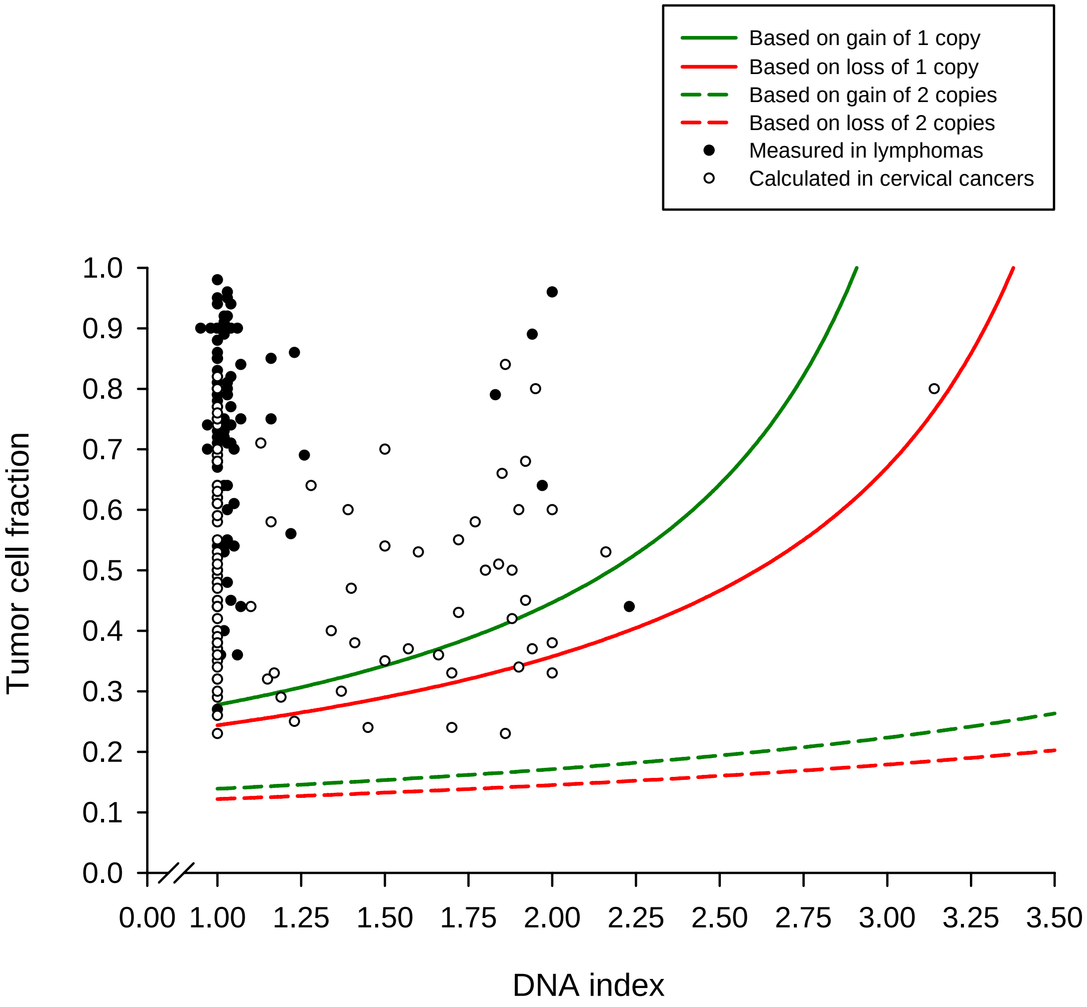

Minimum tumor cell fraction that can be calculated in GeneCount.
The tumor fraction needed for achieving ratio levels of 0.15 above the baseline, assuming a copy number gain (green) or loss (red) of 1 (stippled lines) and 2 (dotted lines) copies, is shown as a function of the DNA index (*DI*). The curves were calculated from Equation 4. Data showing the tumor cell fractions of 94 lymphomas (closed symbols) and 93 cervical cancers (open symbols), as determined by flow cytometry (lymphomas) and estimated by GeneCount from the GLAD ratio levels (cervical cancers) are included.
