# Supplementary material for: GeneCount: genome-wide calculation of absolute tumor DNA copy numbers from array comparative genomic hybridization data
Source: Genome Biol. 2008 May 23;9(5):R86. doi: 10.1186/gb-2008-9-5-r86 (PMC2441472; doi:10.1186/gb-2008-9-5-r86)
Supplement: Additional data file 13 — The tumor cell fraction required for detection of homogeneous copy number changes. [file gb-2008-9-5-r86-S13.pdf]

# Additional data file 13

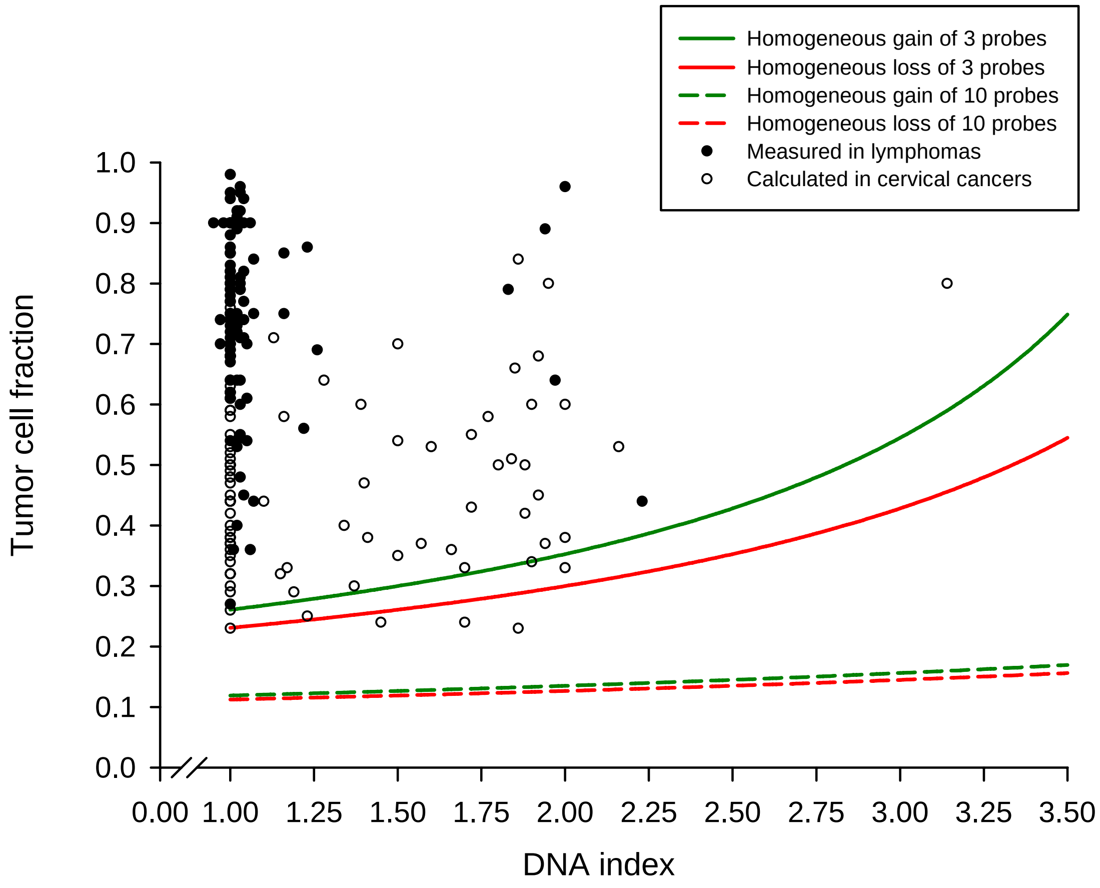


**Tumor cell fraction required for detection of homogeneous copy number changes.**
The tumor fraction needed for statistically significant separation of an aberrant chromosomal region involving more than three (solid lines) or ten (stippled lines) array probes from a region without aberration is shown as a function of the DNA index (*DI*). The curve for gain and loss of one DNA copy (copy number $2 \cdot DI \pm 1$) is shown in green and red color, respectively. A Student t-test and a standard deviation of 0.1 for the log-transformed ratio distributions were used to estimate the curves. Data showing the tumor cell fractions of 94 lymphomas (closed symbols) and 93 cervical cancers (open symbols), as determined by flow cytometry (lymphomas) and estimated by GeneCount from the GLAD ratio levels (cervical cancers) are included.
